# A Metabolomic Analysis of the Sex-Dependent Hispanic Paradox

**DOI:** 10.3390/metabo11080552

**Published:** 2021-08-20

**Authors:** Jeffrey Patterson, Xiaojian Shi, William Bresette, Ryan Eghlimi, Sarah Atlas, Kristin Farr, Sonia Vega-López, Haiwei Gu

**Affiliations:** College of Health Solutions, Arizona State University, Phoenix, AZ 85004, USA; jspatte5@asu.edu (J.P.); xshi49@asu.edu (X.S.); bresette@email.arizona.edu (W.B.); reghlimi@asu.edu (R.E.); satlas@asu.edu (S.A.); kfarr3@cscc.edu (K.F.)

**Keywords:** obesity, macronutrient intake, micronutrient intake, LC-MS/MS, metabolomics, Hispanic paradox

## Abstract

In Mexican Americans, metabolic conditions, such as obesity and type 2 diabetes (T2DM), are not necessarily associated with an increase in mortality; this is the so-called Hispanic paradox. In this cross-sectional analysis, we used a metabolomic analysis to look at the mechanisms behind the Hispanic paradox. To do this, we examined dietary intake and body mass index (BMI; kg/m^2^) in men and women and their effects on serum metabolomic fingerprints in 70 Mexican Americans (26 men, 44 women). Although having different BMI values, the participants had many similar anthropometric and biochemical parameters, such as systolic and diastolic blood pressure, total cholesterol, and LDL cholesterol, which supported the paradox in these subjects. Plasma metabolomic phenotypes were measured using liquid chromatography tandem mass spectrometry (LC-MS/MS). A two-way ANOVA assessing sex, BMI, and the metabolome revealed 23 significant metabolites, such as 2-pyrrolidinone (*p* = 0.007), TMAO (*p* = 0.014), 2-aminoadipic acid (*p* = 0.019), and kynurenine (*p* = 0.032). Pathway and enrichment analyses discovered several significant metabolic pathways between men and women, including lysine degradation, tyrosine metabolism, and branch-chained amino acid (BCAA) degradation and biosynthesis. A log-transformed OPLS-DA model was employed and demonstrated a difference due to BMI in the metabolomes of both sexes. When stratified for caloric intake (<2200 kcal/d vs. >2200 kcal/d), a separate OPLS-DA model showed clear separation in men, while females remained relatively unchanged. After accounting for caloric intake and BMI status, the female metabolome showed substantial resistance to alteration. Therefore, we provide a better understanding of the Mexican-American metabolome, which may help demonstrate how this population—particularly women—possesses a longer life expectancy despite several comorbidities, and reveal the underlying mechanisms of the Hispanic paradox.

## 1. Introduction

Obesity has increased in prevalence among Hispanics in the United States over the last several decades and continues to trend upward [1]. Furthermore, Hispanics suffer from disproportionately higher rates of some comorbidities associated with obesity, such as type 2 diabetes mellitus (T2DM) and metabolic syndrome (MetS) [1]. Despite these recent trends, Mexican Americans possess a 3.8% greater life expectancy than the general population in the United States, a phenomenon referred to as the “Hispanic paradox” [2,3,4].

The current obesity rate for the Hispanic American population is 34.4%, which is significantly higher than the general American population (28.7%) [5,6]. Two major contributors to this upsurge are considered to be low socioeconomic status and a dietary shift from foods with low caloric density to foods with high caloric density, thereby increasing caloric intake [7,8,9]. Despite elevated rates of obesity and T2DM (16.9%), Hispanic Americans, particularly women, demonstrate a resilience towards several metabolic-related illnesses, including cardiovascular disease (8.3%) and cancer incidence (1.6 per 1000 individuals) [10].

The high consumption of fruits, vegetables, and legumes, which are staples in the traditional Mexican diet, has been linked to a lower BMI [11,12]. In contrast, the high consumption of meats, sugar-sweetened beverages, oils, and refined grains—key components of a Western diet—has been associated with a significant increase in BMI [13,14]. Previous studies have shown that exposure to the Western diet after migration to the United States may play a significant role in adverse health outcomes [15,16,17]. Diet and metabolome are closely related, and it is critical to assess mechanisms through the alterations created at the metabolic level. Moreover, the dependence of sex on the metabolome requires consideration. While the influence of sex has been studied extensively in disease and its pathophysiology, more recent studies have begun to further examine its role in chronic metabolic conditions, such as MetS and T2DM [18,19,20]. Sex, BMI status, and dietary intake may all be contributing factors to the resilient metabolome of Mexican Americans.

Metabolomics offers a current physiological report of metabolic pathways through the measurement of metabolites with molecular weight of about <2000 Da [21,22,23,24]. As a powerful detection model, it can ascertain significantly altered major metabolic pathways and shed light on the key metabolic differences associated with nutritional status, varied caloric intake, and chronic conditions such as obesity, cardiovascular disease, and cancer [22,25,26,27,28,29,30]. In particular, large-scale targeted metabolomics has been used to study both BMI status and caloric intake in the general American population. Previous research indicates that there is an association between caloric intake and differing metabolic fingerprints, as people in a caloric deficit show altered fingerprints compared to those in caloric abundance [31,32,33]. Additionally, findings from a large study with non-Hispanic white participants suggest that several metabolites varied significantly among individuals with different BMI status [34]. Furthermore, certain metabolites have been found to not only correlate with current BMI status but also predict future weight gain in Mexican-American women [35]. It is also important to note that the metabolic profiles of men and women have been found to be significantly different in numerous trials, particularly those examining the relationship with BMI [34,36]. Such studies have shown the need to account for the variability in metabolomes between men and women. As a result, metabolomics can be used to identify metabolic profiles through assessing BMI status, caloric intake, and sex in order to help explain the mechanism behind the Hispanic paradox.

In this study, we aim to assess how the metabolome may be associated with the Hispanic health paradox in a cross-sectional study. We utilized a large-scale targeted LC-MS/MS approach to measure the metabolic profiles of 70 normal weight, overweight, and obese Mexican Americans. A two-way ANOVA, OPLS-DA models, and pathway analyses were conducted to identify metabolites and pathways indicative of metabolic resilience that may help explain how sex and BMI differently affect the Hispanic paradox in Mexican Americans.

## 2. Results

### 2.1. Participant Characteristics

The similar anthropometric and biochemical values compared among BMI categories suggest the Hispanic paradox effect in the recruited Mexican Americans in this study. The data are shown in Table 1. Participants included 5 males and 16 females (*n* = 21) representing the normal weight category (BMI < 25), 11 males and 11 females (*n* = 22) representing the overweight category (25 ≤ BMI < 30), and 10 males and 17 females (*n* = 27) representing the obese category (BMI ≥ 30). The total BMI range for males was 23.5–36.8 kg/m^2^, whereas the total BMI range for females was 19.9–45.7 kg/m^2^. The average age of all participants was 36.7 years old, with no significant difference between the three BMI categories. Total cholesterol concentrations were not significantly different (*p* = 0.647), but HDL cholesterol concentrations were significantly lower among participants with an elevated BMI status (*p* < 0.001). The normal, overweight, and obese BMI groups were all normoglycemic (91.18, 97.14, and 93.01 mg/dL, respectively) and had no significant differences in fasting glucose concentrations. With the overweight and obese groups displaying normoglycemia in the presence of hyperinsulinemia, some participants may have been in a fully compensated state. With some expectant similarities and differences between the groups, the participants’ dietary intake was then analyzed.

### 2.2. Food/Nutrient Intake Data

Male and female Mexican Americans in different BMI groups had different nutrient intake. There were no significant differences in any intake metric, including total energy, food groups, macronutrients, and micronutrients, among males in different BMI groups (Table 2 and Table S1). Normal-weight men consumed 2849.2 ± 1416.4 kcal/day, overweight men consumed 2593.1 ± 1122.8 kcal/day, and obese men consumed 3887.6 ± 2783.6 kcal/day. In contrast, obese women ingested significantly more energy (3325.6 ± 1294.9 kcal/day vs. 1920.1 ± 682.8 kcal/day; *p* < 0.001), total protein (*p* < 0.003), red meat (*p =* 0.001), white meat (*p* = 0.025), and fish (*p* = 0.017) compared to their normal-weight counterparts. No significant differences were observed between the normal and overweight groups of women (Table 3 and Table S2).

As a percentage of calories, Table S3 shows that carbohydrates, fat, and protein were not significantly different between normal-weight men, overweight men, and obese men, with carbohydrates consisting of 49%, 52%, and 52% of total calories and fat consisting of 32%, 31%, and 31% of total calories, respectively (Table S3 and Figure S1). However, obese women proportionally ate significantly less carbohydrates and more fat compared to their normal weight and overweight counterparts (*p <* 0.05), with normal weight, overweight, and obese women consuming 55%, 54%, and 50% of their calories from carbohydrates and 28%, 30%, and 33% of their calories from fat, respectively (Table S4 and Figure S1).

### 2.3. Metabolomic Analysis

The effects of sex and BMI on the metabolome were examined through a two-way ANOVA, and 23 metabolites were found to be significant across the three groups. For the BMI group, pipecolic acid (*p* = 0.002), creatinine (*p* = 0.003), acetylcarnitine (*p* = 0.004), 2-pyrrolidinone (*p* = 0.007), and TMAO (*p* = 0.014) were among the most significant metabolites. Glucose (*p* = 0.005) and succinate (*p* = 0.032) were the only two significant metabolites in the Sex group. Pregnenolone sulfate (*p* = 0.016), fructose (*p* = 0.021), phenylpyruvic acid (*p* = 0.023), glyceric acid (*p* = 0.039), and acetylornithine (*p* = 0.046) were all significant in the Sex and BMI group (Table 4). A Tukey’s HSD correction was also performed to examine the differences in significant metabolites across the three BMI groups (Table S5). Acetylcarnitine, asparagine, creatinine, glutamic acid, pipecolic acid, cytidine, picolinic acid, kynurenine, and 2-aminoadipic acid were upregulated in the normal BMI group in comparison to the overweight group. 2-pyrrolidinone was also found upregulated in the normal group in contrast with the obese group, while decanoylcarnitine was upregulated in the obese group, but not in the normal weight population. Acetohydroxamic acid, TMAO, creatinine, glutamic acid, cytidine, leucic acid, D-galacturonic acid, picolinic acid, and 2-aminoadipic acid were found to be downregulated in the overweight group compared to the obese group. Both succinate and glucose/galactose were determined to be downregulated in men. Line plots of the significant metabolites and their mean abundances and standard deviations were also conducted to reflect the alterations across the three BMI groups (Figure S2). In order to further investigate the differences in the male and female metabolomes, additional assessments were conducted.

A correlation heatmap of the two-way ANOVA significant metabolites and subject demographics was generated with Pearson’s *r* (Figure S3 and Table S6). The correlation cutoff was 0.5, and all subjects were included. TMAO and acetohydroxamic acid demonstrated the largest correlation (*r* = 0.995, *p* ≤ 0.001), while glucose and fructose shared the second strongest correlation (*r* = 0.879, *p* ≤ 0.001). Isoleucine also displayed robust correlations with kynurenine (*r* = 0.826, *p* ≤ 0.001), glutamic acid (*r* = 0.790, *p* ≤ 0.001), and creatinine (*r* = 0.647, *p* ≤ 0.001). Picolinic acid had similar correlations with kynurenine (*r* = 0.684, *p* ≤ 0.001), glutamic acid (*r* = 0.518, *p* ≤ 0.001), creatinine (*r* = 0.533, *p* ≤ 0.001), and 2-aminoadipic acid (*r* = 0.580, *p* ≤ 0.001). Kynurenine, glutamic acid, and creatinine were all significantly correlated with 2-pyrrolidinone (*r* = 0.669, *p* ≤ 0.001; *r* = 0.601, *p* ≤ 0.001; *r* = 0.533, *p* ≤ 0.584; respectively). Asparagine was also similarly correlated with kynurenine (*r* = 0.606, *p* ≤ 0.001), glutamic acid (*r* = 0.644, *p* ≤ 0.001), and creatinine (*r* = 0.513, *p* ≤ 0.001).

**Figure 1:**
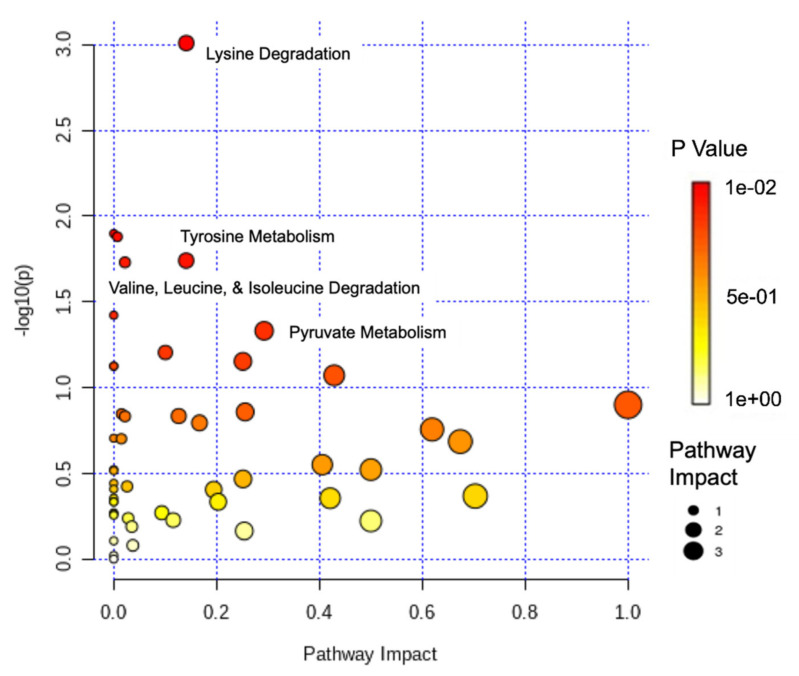
Log-transformed pathway analysis of sex and the metabolome. Data is plotted as significance (−log_10_(*p*)) versus pathway impact.

**Figure 2:**
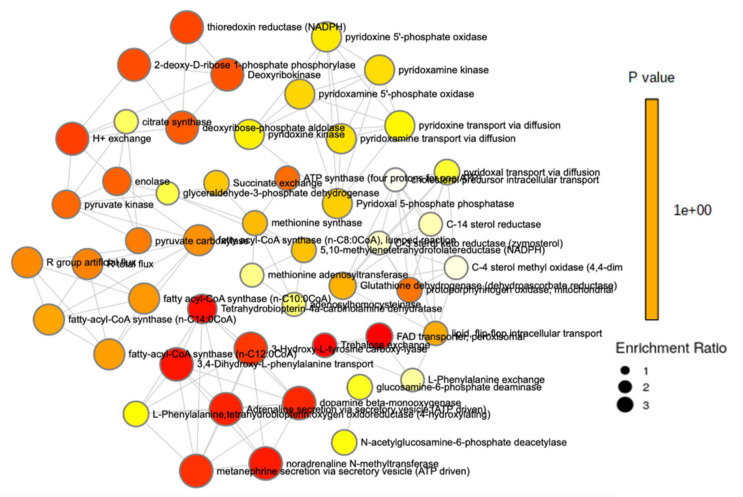
Enrichment analysis of sex and the metabolome.

Pathway and enrichment analyses were performed to examine the impact of sex on the metabolome. Lysine degradation had the most significance (*p* < 0.001) and the second largest impact, followed by vitamin B_6_ metabolism (*p* = 0.012) and pantothenate and CoA biosynthesis (*p* = 0.013). Tyrosine metabolism and valine, leucine, and isoleucine degradation both had a significance of *p* = 0.012, while valine, leucine, and isoleucine biosynthesis was also significant (*p* = 0.029). Pyruvate metabolism demonstrated a significance of *p* = 0.046 (Figure 1 and Table S7). An enrichment analysis of more than 900 metabolic sets related to dysfunctional enzymes showed that perixosomal FAD transporters (*p* = 0.016), trehalose exchange (*p* = 0.017), thioredoxin reductase (*p* = 0.023), deoxyribokinase (*p* = 0.023), and fatty acyl-CoA synthase (*p* = 0.034) were among the most significant (Figure 2 and Figure S4).

**Figure 3:**
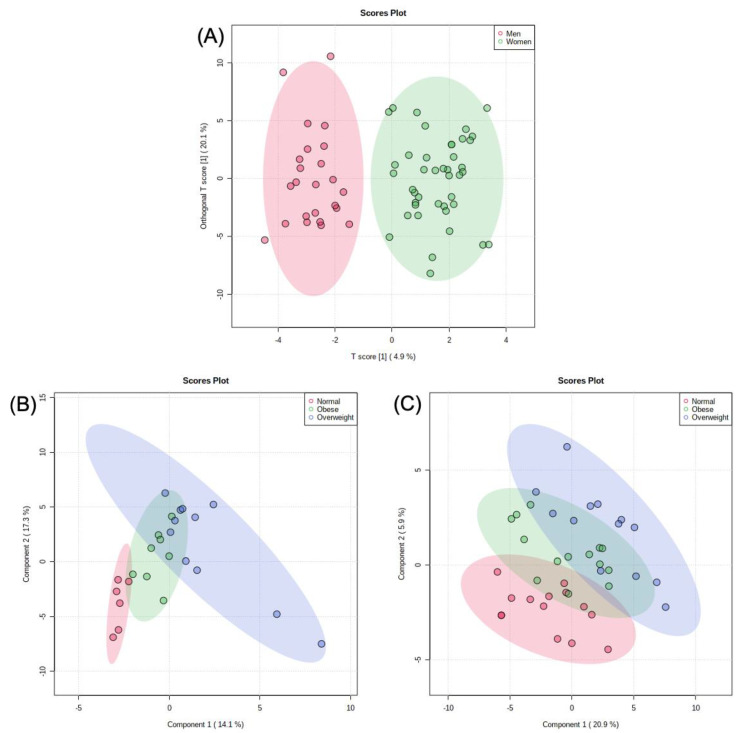
(**A**) Log-transformed OPLS-DA model of men’s metabolites vs. women’s metabolites. (**B**) Log-transformed PLS-DA model comparing men’s BMI using their metabolic profiles. (**C**) Log-transformed PLS-DA model comparing women’s BMI using their metabolic profiles.

An independent Student’s *t*-test was used to compare metabolites of male and female participants, with 24 metabolites being significantly different. Males had a higher concentration of proline, betaine, L-alloisoleucine, isoleucine, 2-methyl-2-oxovaleric acid, glutamic acid, citraconic acid, 4-methyl-2-oxopentatonic acid, 2/3-aminoisobutyric acid, hypoxanthine, 2-hydroxyphenylacetic acid, leucic acid, picolinic acid, tyrosine, 3-phenyllactic acid, phenylpyruvic acid, and 2-aminoadipic acid. Meanwhile, females had a higher concentration of myristic acid, 2,3-dihydroxybenzoic acid, protocatechuic acid, creatine, 9-octadecyonic acid, and lauric acid (Table S8). A log_10_-scaled OPLS-DA model showed significant separation between men and women (Figure 3A). A log_10_-scaled PLS-DA model was used for both men and women separately to assess changes in metabolic profiles among differing BMIs (Figure 3B,C). After accounting for BMI status, the study participants were then stratified for overall daily intake.

**Figure 4:**
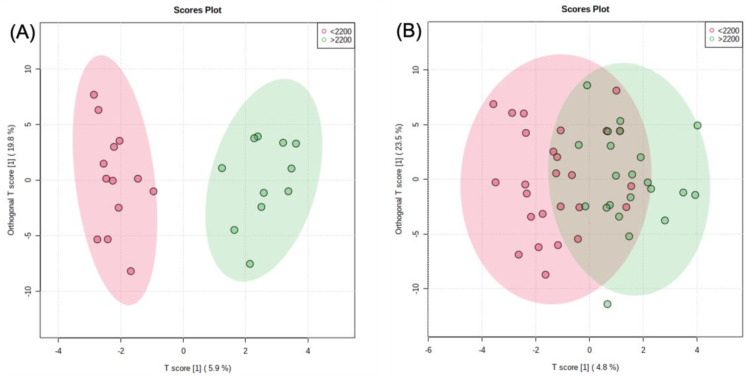
Log-transformed OPLS-DA models distinguishing metabolic phenotype of a low calorie (<2200 kcal/day) group and a high calorie (>2200 kcal/day) group. The two score plots represent (**A**) men and (**B**) women.

Both men and women were split into two groups based on caloric intake: <2200 kcal/day—the low caloric intake group, and >2200 kcal/day—the high caloric intake group. An independent Student’s t-test was utilized, and a log-transformed OPLS-DA model was constructed. Three metabolites were significantly greater in men in the low caloric intake group, including proline (*p* = 0.022), pyruvate (*p* = 0.016), and leucic acid (*p* = 0.033). Two metabolites were significantly greater in men in the high caloric intake group, including 3-hydroxykynurenine (*p* = 0.044) and erythrose (*p* = 0.014). The OPLS-DA model shown in Figure 4A for men showed clear separation with no overlap, meaning the metabolic fingerprint of men with a lower caloric intake was significantly different than that of men with an increased caloric intake. There was much more overlap between the high calorie group and the low-calorie women’s group when using the log-transformed OLPS-DA model shown in Figure 4B. The low calorie group had three significantly increased metabolites, including creatinine (*p* = 0.028), cytidine (*p* = 0.042), and pantothenic acid (*p* = 0.024), and the high calorie group had three significantly increased metabolites, including valine (*p* = 0.048), norvaline (*p* = 0.044), and carnitine (*p* = 0.021).

A corrected multivariate analysis of variance (MANOVA) was conducted for men’s and women’s caloric intake to control for body weight and physical activity. After applying the covariates, methylguanidine (*p* = 0.004) and 2-hydroxyphenylacetic acid (*p* = 0.035) were identified as significant in the men’s group, while stearic acid (*p* = 0.002), nonadecanoic acid (*p* = 0.011), malic acid (*p* = 0.026), and dimethylglycine (*p* = 0.032) were found to be significant in the women’s group.

## 3. Discussion

The Hispanic paradox is a term used to describe how Mexican Americans demonstrate an increased life expectancy despite lower socioeconomic status and increased prevalence of obesity and its comorbidities when compared with the general population [2,3,4]. A potential explanation is that Mexican Americans possess a metabolome that leans towards health-promoting rather than health-deteriorating, regardless of weight. However, the mechanisms behind the impact of sex, BMI status, and dietary intake on the metabolic phenotype of Mexican Americans are largely unknown. Genetics and lifestyle both play a significant role in the pathogenesis of obesity and its comorbidities. Dietary intake has been shown to be one of the largest modulators of epigenetic changes and the subsequent onset of obesity and disease [11,12,13]. The current diet of Mexican Americans has been transitioning over the past several decades from a diet centered around whole-plant foods, calorically low but nutrient dense, to high caloric and low-nutrient-dense foods [11,12,13]. A current, long-term epidemiology study, The Hispanic Community Health Study/Study of Latinos (HCHS/SOL), has examined the frequent occurrence of chronic disease in 16,000 adult Hispanic and Latino Americans, spanning back to 2006. The recent study data have found that 80% of men and 71% of women have at least one risk factor for cardiovascular disease (CVD), which highlights the effects of the paradox [37]. In addition to overall intake, it is prudent to consider the effects of sex on metabolism and disease. Recent studies have begun to investigate how sex may play a role in risk, pathophysiology, complications, and treatments in metabolic diseases, such as MetS and T2DM [18,19,20]. Investigating the dependence of sex with BMI status and energy intake on the Mexican American metabolome may help uncover the significant pathways of the Hispanic paradox.

With a cross-sectional analysis of dietary intake and large-scale targeted metabolomics, we stratified 70 Mexican Americans in the Phoenix metropolitan area into men and women with three BMI categories to find a relationship between sex, BMI, total energy intake, and serum metabolite concentrations. After examining the participant baseline data, the statistical results supporting the paradox hold accurate (Table 1). There are many unexpected similarities between the three BMI groups, such as very little change in systolic and diastolic blood pressure, total cholesterol, and LDL cholesterol among the groups. Outside of this study population, one would anticipate a larger discrepancy in metabolic function.

A two-way ANOVA examining the effects of sex and BMI on the Mexican American metabolome was employed. Results indicated differences in 23 metabolites among the three groups. Most notably, in the BMI group, acetylcarnitine (*p* = 0.004), 2-pyrrolidinone (*p* = 0.007), 2-aminoadipic acid (*p* = 0.019), and kynurenine (*p* = 0.032) had demonstrated significance and were upregulated in the normal group (Table 4 and Table S5 and Figure S2). In recent studies, both acetylcarnitine and 2-pyrrolidinone have been linked as potential factors in stabilizing blood glucose levels and diet-induced obesity. As an enzyme found within the mitochondrial matrix, carnitine acetyltransferase is tasked with the conversion of acetyl-CoA to acetylcarnitine. It is a potentially vital enzyme in increasing pyruvate dehydrogenase (PDH) activity, which stimulates glucose oxidation and regulates blood glucose [38]. With increased acetylcarnitine concentrations, PDH activation may be expected. Interestingly, 2-pyrrolidinone was one of the only metabolites to be downregulated in the obese group. Its anorectic effects and derivatives were examined for their influence on α-adrenoreceptors in obese mice. It was found that their ability to serve as an antagonist to α2-adrenoreceptors resulted in reduced body weight through decreased food intake and blood glucose levels with improved lipolysis and thermogenesis [39]. In addition, 2-aminoadipic acid has been shown to be not only a potential biomarker for T2DM, but also a protective agent against T2DM and obesity [40]. With an opposing effect, the upregulated kynurenine pathway has been highly associated with endothelial dysfunction and cardiovascular disease due to its role in regulating inflammation [41]. Moreover, kynurenine induces immunosuppression by activating the AhR receptor, which inhibits T cells from destroying cancer cells [42]. The protective effects of the previously mentioned metabolites appear to aid in countering the well-researched kynurenine pathway and its role in metabolic disease.

In addition, multiple other metabolites were found to be elevated in the lower BMI classes. A non-essential amino acid, asparagine (*p* = 0.018), facilitates glycoprotein synthesis and aids in the excretion of ammonia [43]. It has also been shown that intracellular asparagine regulates cellular uptake of serine, arginine, and histidine and, as a result, mTORC1 activation and protein synthesis [44,45]. Interestingly, a previous study examining BMI and weight gain in Mexican American women found similar results and reduced asparagine levels with increased weight [35]. Additionally, as a metabolite of tryptophan metabolism and product of the kynurenine pathway, picolinic acid (*p* = 0.044) has been thought to have anti-proliferative, immunological, and neuroprotective effects [46]. Furthermore, as the main component of pectin, D-galacturonic acid (*p* = 0.042) has been investigated for its use as an anti-inflammatory agent in intestinal cell permeability and was found to attenuate inflammation as a result of poor function [47].

Elevated in the normal weight group, but significantly increased in the obese group, TMAO (*p* = 0.014) has been of recent interest to researchers. Several studies have investigated its role in insulin resistance and cancer for new pharmacological targets [48,49]. The consumption of certain nutrients, such as choline and carnitine, from red meat results in the production of trimethylamine by the intestinal bacteria, which is then converted to TMAO in the liver. As a metabolite produced by the gut microbiome, elevated circulating levels of TMAO have been shown to be correlated with increased risk of clotting conditions, such as a stroke and heart attack [50]. Due to the large prevalence in cardiovascular disease and T2DM, it has become a well-researched predictor of obesity in adults [51,52].

In the Sex group, the glycolysis substrate, glucose (*p* = 0.005), and TCA cycle intermediate, succinate (*p* = 0.032), were the only significant metabolites and were downregulated in men, which may highlight a difference in energy usage between Mexican American men and women. In addition, reduced glucose levels may be a result of increased insulin resistance [53]. Similarly in the Sex and BMI group, the simple sugar and potential energy source, fructose (*p* = 0.021), was identified. Many studies have identified the potential harmful effects of ingesting high quantities of fructose. Due to its inability to promote a satiety comparable to that of glucose, fructose intake results in higher consumption of food and increased risk of obesity and insulin resistance [54]. It should be noted that in a fasting state, energy substrates and storage molecules, such as ketone bodies and glycogen may play a role in the measured blood glucose and insulin levels. In the same group, an interesting metabolite was revealed, pregnenolone sulfate (*p* = 0.016). Pregnenolone sulfate is a neurosteroid that aids in the regulation of gene expression. A recent study showed its involvement in improving cognitive ability, reducing depression, and promoting neuronal cell survival [55]. Phenylpyruvic acid (*p* = 0.023) was also identified in the Sex and BMI group and is a derivative of phenylalanine metabolism. A close product found in the plant rooibos and commonly used for herbal tea, phenylpyruvic acid-2-O-β-D-glucoside is a protective agent against heart disease and hyperglycemia-induced oxidative stress. It is thought that the mechanism of action is the inhibition of cellular apoptosis induced by endoplasmic reticulum stress, mitochondrial depolarization, impairment of substrates, and insulin resistance [56]. A discrepancy among the harmful and protective metabolites in the Sex and BMI group is evident, potentially identifying differing metabolomes among men and women.

An additional analysis of the significant metabolites was conducted to examine potential correlations between the metabolites (Figure S3 and Table S6). While the literature is lacking in the association of TMAO and acetohydroxamic acid, the two metabolites demonstrated the strongest correlation (*r* = 0.995, *p* ≤ 0.001). As previously mentioned, TMAO has extensive studies demonstrating its presence in obesity, cardiovascular disease, and T2DM, and it has also been shown to be a uremic retention solute in kidney dysfunction [57]. Acetohydroxamic acid, a urease inhibitor, has long been used to treat renal stones and urinary infections [58]. While there is no literature on the clear association of TMAO and acetohydroxamic acid, it may be hypothesized that the potential correlation between the two compounds is urea and compromised kidney function, typically observed in T2DM-related kidney disease. It is also expected that two simple sugars, glucose and fructose (*r* = 0.879, *p* ≤ 0.001), which are both involved in energy metabolism, would share such a high correlation. Recent studies have concluded that low doses of fructose are cleaved into three-carbon molecules and converted into glucose by the small intestine. However, high doses of fructose saturate the enzyme, ketohexokinase, and are sent to the liver to be converted to glyceraldehyde-3-phosphate for glycolysis [59]. A separate study utilizing isotopic tracers postulated that the fructose to glucose mean conversion rate was 41% three to six hours after intake and confirmed the hyperlipidemic outcomes of excess fructose ingestion [60]. With the high rates of obesity in Mexican Americans, high doses of fructose should not be surprising, but it does not seem to result in the same hyperlipidemia profiles as other populations.

The association of kynurenine and metabolic disease has been mentioned, but its robust correlation with isoleucine (*r* = 0.826, *p* ≤ 0.001) and other amino acids is an intriguing concept. Multiple amino acids, particularly isoleucine, have been proven able to significantly reduce the formation and concentrations of the tryptophan metabolite, kynurenine, and its derivative, kynurenic acid. It has been highlighted that large neutral amino acid transporters (LATs), which are vital for the production of kynurenic acid, have a much higher affinity for branched-chain amino acids (BCAAs) [61]. While BCAAs have their own correlation with T2DM, their ability to reduce the pro-atherogenic properties of kynurenine may help shed light on the paradox, which involves high levels of T2DM but lower than expected cardiovascular disease. Additionally, isoleucine was strongly associated with glutamic acid (*r* = 0.790, *p* ≤ 0.001), a derivative of glutamine. This is because BCAAs, such as isoleucine, are the primary nitrogen source in the synthesis of glutamine.

The neuroprotective metabolite, picolinic acid, is synthesized through the kynurenine pathway and thus their identified correlation (*r* = 0.684, *p* ≤ 0.001) is expected. Kynurenine was also found to have a significant association with 2-pyrrolidinone (*r* = 0.669, *p* ≤ 0.001). The anorectic effects of 2-pyrrolidinone on α2-adrenoreceptors have been noted [39], which may contribute to this correlation with kynurenine. Asparagine was also found to have a similar correlation with kynurenine (*r* = 0.606, *p* ≤ 0.001). Asparagine is important in glycoprotein synthesis, ammonia carrying, and providing carbon backbones for the TCA cycle, which indicates that these biological processes could be involved in the mechanisms of the Hispanic paradox.

Pathway and enrichment analyses were also performed to better investigate the potential impact of sex on metabolic pathways in Mexican Americans (Figure 1 and Figure 2). The results indicate several pathways linked to cardiovascular disease, insulin resistance, and T2DM. Lysine degradation and tyrosine metabolism had the highest level of impact and significance (*p* < 0.001 and *p* = 0.018, respectively). As a metabolic intermediate of lysine metabolism, 2-aminoadipic acid and its protective effects have been previously discussed [40]. In addition, a systemic review also showed the effects of lysine and its residues on critical metabolic reactions pertaining to cardiometabolic health and T2DM [62]. In another recent study, the effects of plasma tyrosine levels on T2DM risk were investigated in the presence of low HDL-C. It was determined that reduced HDL-C and elevated tyrosine levels were associated with an increase in T2DM risk [63]. The analysis also revealed a significant alteration in BCAA metabolism, particularly the biosynthesis and degradation of BCAAs (Table S7). It is well documented that circulating levels of BCAAs are associated with the incidence of insulin resistance, cardiometabolic diseases, and obesity [64,65,66]. With a high prevalence of T2DM and obesity in Hispanic Americans, high levels of BCAAs would be expected.

In order to examine the difference in the metabolic profiles of men and women, a log-transformed OPLS-DA model was generated (Figure 3A). The OPLS-DA model demonstrated good separation between the groupings and displayed the potential differences in the metabolomes of both sexes. A log-transformed PLS-DA model was also created for normal-weight, overweight, and obese men and women (Figure 3B,C). While the BMI model produced less clear results, there were identifiable trends among the normal-weight, overweight, and obese groups.

After investigating the influence of sex and BMI status, the subjects were stratified for sex and energy intake. An OPLS-DA model did reveal clear separation in relation to relative metabolite concentrations (Figure 4A). The low-energy intake group (<2200 kcal/day) had significantly higher plasma concentrations of proline, pyruvate, and leucic acid. It has been previously shown that plasma amino acid levels, including proline, increase in times of lower caloric intake and weight loss [67]. Intriguingly, one study found increased plasma pyruvate concentration was associated with decreased weight gain [68]. Leucic acid is an intermediate metabolite in the degradation of lysine and is considered to be an anti-catabolic metabolite [69]. The high energy intake group (>2200 kcal/day) had significantly higher 3-hydroxykynurenine and erythrose metabolites. 3-hydroxykynurenine is an intermediate in the degradation of tryptophan, while erythrose is a 4-carbon aldose that is readily converted into erythronate. Previous studies have shown an increased plasma concentration of both of these to be linked to obesity [70,71].

While the female low (<2200 kcal/day) and high (>2200 kcal/day) energy consumption groups displayed some separation in their metabolic profiles (Figure 4B), the more interesting finding is that females showed very little separation in comparison with the male groups. Despite differing caloric intake, the female metabolome displayed resiliency toward a higher energy intake. Six metabolites were significantly different between the low and high energy intake groups. The low energy intake group had significantly higher concentrations of creatinine, cytidine, and pantothenic acid. Creatinine is a waste product from the breakdown of muscle and is a reliable indicator of kidney function. It should be noted that creatinine concentrations have been proven to be significantly affected by large quantities of meat ingestion. In a previous study examining diabetes-related kidney disease, it was determined that after a 12 h fast, the effect of meat consumption had been normalized in all subjects [72]. A recent study has also shown that low circulating levels of creatinine may be a risk factor for T2DM in both sexes [73]. As a pyrimidine, cytidine is integrated into nucleic acids and needed for the critical phosphatidylcholine (PC) and phosphatidylethanolamine (PE) pathways. Imbalances in PC:PE ratios have been linked to the advancement of disease due to their impact on energy metabolism [74]. Pantothenic acid, also known as vitamin B5, is used as a precursor to coenzyme A (CoA). CoA has a whole host of uses in the metabolism of lipids, proteins, and CHOs, and an increased CoA metabolite level would indicate that there is a decrease in energy intake [75].

In contrast with Mexican American women with low energy consumption, the female high energy intake group had significantly greater concentrations of valine, norvaline, and carnitine. Valine is an essential BCAA that has been shown to increase energy expenditure in humans and is generally not associated with weight gain [76]. Norvaline is an isomer of valine and would also be expected to increase based on increased valine plasma concentration [77]. As previously identified with the pathway analysis, valine, leucine, and isoleucine biosynthesis and degradation pathways were significant. A study examining BCAA metabolic signatures in lean and obese non-Hispanic Americans also found elevated concentrations of valine, leucine, and isoleucine in obese men and women [78]. As a dipeptide made from lysine and methionine, carnitine plays an essential role in shuttling fatty acids for β-oxidation in the mitochondria and has been associated with a lower weight status [79]. Additionally, the previous two-way ANOVA revealed the significance of its derivative, acetylcarnitine, among the BMI groups and its ability to regulate blood glucose via PDH stimulation [80]. While these three metabolites are typically associated with energy consumption, their prevalence in the non-Hispanic male and female obese group may demonstrate a metabolic shift that drives a healthy metabolome in Mexican American women.

Furthermore, a corrected MANOVA model for caloric intake in men and women was generated to consider the effects of current body weight and physical activity (Table 5). In men, both methylguanidine (*p* = 0.004) and 2-hydroxyphenylacetic acid (*p* = 0.035) were found to be significant. The typical mechanism in which methylguanidine is formed is through the oxidation of creatinine, which occurs in a diet rich in boiled beef [81,82]. Both methylguanidine and 2-hydroxyphenylacetic acid are common urea waste products, which may signify a diet centered on red meat consumption and compromised kidney function in the obese group.

For the female group, the saturated fatty acids, stearic acid and nonadecanoic acid, were among the most significant. Despite the well-known harmful effects of saturated fatty acids, stearic acid (*p* = 0.002) behaves more similarly to an unsaturated fatty acid. Studies have shown that stearic acid is able to improve mitochondrial function through mitofusin-2, a cellular metabolism facilitating protein, and lipid metabolism [83,84]. A similarly intriguing metabolite is nonadecanoic acid (*p* = 0.011), which has protective effects against cancer. Identified in the two-way ANOVA, it was one of the few compounds to display significantly reduced levels in the obese group. It is abundantly found as a phytochemical within the reishi mushroom (Ganoderma lucidum); more typical but less plentiful sources of nonadecanoic acid are vegetable oils [85]. Dietary choices and cooking methods may explain the rise in the overweight group, but a decline for the obese group. The natural sources of malic acid (*p* = 0.026) are typically vegetables, berries, cherries, and citrus fruits; more recently, it has been incorporated into popular fruit vinegar drinks for its antioxidant properties and taste [86]. Commonly found in beans, a staple of the Hispanic diet, dimethylglycine (*p* = 0.032) is known to exert beneficial effects. It has been proven to aid in the prevention of oxidative stress, mitochondrial dysfunction, and T2DM, while improving energy through increased nutrient digestibility and glucose metabolism [87,88,89]. In contrast with the identified metabolites in the male group, the female metabolome continues to demonstrate how it may be more resilient to existing comorbidities.

Despite our findings, the study did contain several limitations. A cross-sectional study design was employed, which cannot allow for the analysis of variations in dietary intake and BMI. In addition, the participants were a homogenous sample of healthy adults, with a relatively small sample size: *n* = 21 subjects for the normal weight group, *n* = 22 subjects for the overweight group, and *n* = 27 subjects for the obese group. Additionally, there was an over-representation of women in the normal group. The subjects’ diets were also assessed with a food frequency questionnaire, and the findings were reliant on self-reported data.

## 4. Materials and Methods

### 4.1. Participants

Generally healthy adults (age 18–60 y) of Mexican descent were recruited from the Phoenix Metropolitan area between March and June 2010 through flyers, advertisements in Spanish media, and word of mouth. Prospective participants were asked to complete a screening questionnaire by phone to ensure they met the inclusion criteria. Participants were excluded for any of the following reasons: (a) pregnancy or breastfeeding; (b) known chronic conditions (e.g., diabetes, heart disease, cancer, renal disease, hepatitis, uncontrolled thyroid disease); (c) use of cholesterol-lowering medications; (d) history of difficult vein access or fear of needles; (e) body weight lower than 110 pounds (50 kg); (f) restrictive dietary regime (e.g., veganism, very low carbohydrate diet); and (g) participation in any other research study in which diet was being assessed or manipulated. A total of 75 qualifying participants attended a study visit after an overnight fast (12 h) for the collection of anthropometric measurements, blood pressure, and a fasting blood sample as well as for completion of a study survey and a food frequency questionnaire. The present analysis included 70 participants (26 males, 44 females) from whom there was sufficient stored plasma to perform large-scale targeted liquid chromatography tandem mass spectrometry (LC-MS/MS) analysis. The study was approved by the Institutional Review Board at Arizona State University, and all participants provided written consent prior to study participation. All study documents were available in English and Spanish, and participants could use their language of preference.

### 4.2. Anthropometric Assessment

Upon arrival to the study visit and after confirming fasting status, participants were asked to empty their bladder prior to collecting anthropometric measurements (all in triplicate). Weight (in kilograms) and body fat percentage were measured using a Tanita body composition analyzer (Tanita Corporation of America, Arlington Heights, IL, USA). Height was measured in centimeters using a wall-mounted stadiometer. Waist and hip circumferences were measured following standardized protocols using a flexible measuring tape. BMI was calculated as weight divided by height in meters squared (kg/m^2^). Blood pressure was measured from the non-dominant arm following a 5 min rest using an electronic sphygmomanometer (Intelli-Sense Blood Pressure Monitor HEM-907-XL, Omron Healthcare, Kyoto City, Japan).

Normal weight was defined as having a BMI between 18.5 and 24.99 kg/m^2^, overweight was defined as having a BMI between 25 and 29.99 kg/m^2^, and obese was defined as having a BMI equal to or greater than 30 kg/m^2^.

### 4.3. Biological Markers Assessment

A 40 mL fasting blood sample was collected from the antecubital vein into evacuated tubes and centrifuged at 1100× *g* at 4 °C for 20 min. EDTA-plasma was separated, stored at −80 °C, and analyzed with a Cobas C111 auto analyzer (Roche, Basel, Switzerland). The biomarkers were total cholesterol, HDL, LDL, triglycerides, hsCRP, and glucose. Insulin was measured via Radioimmunoassay (Millipore, Darmstadt, Germany). The Homeostatic Model of Insulin Resistance (HOMA-IR) was calculated with the formula of fasting insulin (mU/mL) × fasting glucose (mg/dL)/405 [90].

### 4.4. Diet/Lifestyle Assessment

Diet was assessed using the Southwestern Food Frequency Questionnaire, a validated bilingual (English/Spanish) questionnaire that includes food items commonly consumed by Hispanics in the Southwestern U.S. [91,92,93]. Output variables derived from this questionnaire include intake amount of macro and micronutrients, amino acids, fatty acids, cholesterol, and carotenoids.

### 4.5. Sample Preparation for Metabolomic Analyses

The following method was modeled after previous protocols [23,24,30,94,95,96]. The metabolic pathways under investigation were selected from >30 metabolic pathways of important biological significance, including glycolysis, TCA cycle, amino acid metabolism, fatty acid metabolism, etc. Initially, plasma samples (50 μL each) were homogenously mixed with a solution containing 500 μL MeOH and 50 μL internal standard solution (1× PBS containing 1.81 mM L-lactate-^13^C_3_ and 142 μM L-Glutamic acid-^13^C_5_). Subsequently, the mixtures underwent vortexing for 5 s and were stored at −20 °C for 20 min, before being centrifuged at 14,000 RPM for 10 min. A total of 450 μL of supernatant was then extracted from each original sample container and separately transferred into a new 2 mL Eppendorf vial and immediately dried in a CentriVap Concentrator at 37 °C for 120 min. The newly dried samples underwent reconstitution in 150 μL of 40% PBS/60% acetonitrile (ACN) and were promptly vortexed until 5 s had passed. Each sample was then centrifugated at 14,000 RPM for 10 min, and 100 μL of supernatant was extracted from the 2 mL Eppendorf vial and transferred into an LC vial for LC-MS/MS analysis. The remaining supernatant (50 μL) from each sample was pooled together in order to create a quality-control (QC) sample. During the LC-MS/MS assay, the QC sample was analyzed once every 10 study sample runs.

### 4.6. LC-MS/MS Analyses

Targeted metabolic LC-MS/MS assay of each plasma sample was conducted using an Agilent Technologies 1290 UPLC-6490 Triple Quad MS system (Santa Clara, CA, USA). Liquid chromatography separation was achieved using an Xbridge^®^ BEH Amide column (2.5 μm, 2.1 × 150 mm; Waters, Milford, MA, USA) at a temperature of 40 °C. Hydrophilic interaction liquid chromatography (HILIC) and multiple reaction monitoring (MRM) methods were applied to quantify 180 metabolites utilizing the measured retention time from their respective standards.

The sample injection volume was calibrated to 4 μL for positive ionization mode and 10 μL for negative ionization mode, with a flow rate of 0.3 mL/min. The stock solution contained 10 mM NH_4_OH and 10 mM NH_4_OAc in ACN. The mobile phase of the LC system consisted of solvent A (ACN: stock = 5:95) and B (ACN: stock = 95:5) for both positive and negative ionization modes. The LC gradient was identical for both positive and negative ionization modes. Initially, each sample underwent a 1.0 min isocratic elution of 10% A. The LC gradient percentage of Solvent A was then linearly increased to 60% at *t* = 11 min. At *t* = 15.5 min, the percentage of A was consequently reduced to 10% in preparation for the next injection. The net experimental time for each individual sample injection was 30 minutes.

The QQQ mass spectrometer was performed under the following conditions: the capillary voltage for positive and negative ionization modes was 3.5 kV, gas temperature was set at 175 °C, and nitrogen flow rate was continuously 15 L/min; meanwhile, the nebulizer was calibrated to 30 psi, the sheath gas was 225 °C, and the flow rate of the sheath gas was set at 11 L/min.

### 4.7. Statistical Analysis

A coefficient of variation (CV) of less than 20% and a collective 80% peak intensity of greater than one thousand for each metabolite were employed. Any missing values were omitted from analysis, and metabolites fulfilling these criteria (*n* = 109) were selected for analysis. A false discovery rate (FDR) cutoff of 0.05 was applied. BMI was split into three categories: normal weight (<25 BMI), overweight (25 < BMI < 30), and obese (BMI > 30). Two-way analysis of variance (ANOVA) was employed to analyze sex, BMI, and the metabolome. A Tukey’s HSD test for post hoc analysis was performed for the ANOVA model. A Multivariate ANOVA (MANOVA) was produced for caloric intake testing, with physical activity and body weight as covariates, using SPSS 22.0 (SPSS Inc., Chicago, IL, USA). Physical activity was scored according to the Stanford Brief Activity Survey and the Rapid Assessment of Physical Activity. Independent Student’s *t*-tests were used to measure caloric intake in men and women, as well as the mean nutrient consumption between each BMI group, using Microsoft Excel (Redmond, WA, USA). Partial least squares-discriminant analysis (PLS-DA), orthogonal PLS-DA (OPLS-DA), pathway analysis, enrichment analysis, and Pearson’s *r* correlation models were log-transformed and run between groups using MetaboAnalyst software [97].

## 5. Conclusions

With the goal of studying the Hispanic paradox, the metabolic profiles among Mexican American men and women were investigated through LC-MS/MS-based large-scale targeted metabolomics analysis. When stratified for BMI and caloric intake, the female metabolome showed substantial resistance to alteration. Additionally, pathway and enrichment analyses revealed significantly altered profiles and metabolism, which have been commonly linked with metabolic conditions such as CVD, insulin resistance, and T2DM. The understanding of these pathways and their impact may help explain how Mexican Americans, particularly women, possess a longer life expectancy despite comorbidities and reveal the underlying mechanisms of the Hispanic paradox.

## Figures and Tables

**Table 1:** Baseline data between normal, overweight, and obese participants.

Measurement	BMI Categories			*p*-Value		
	Normal	Overweight	Obese	Normal vs. Overweight	Normal vs. Obese	Overweight vs. Obese
Gender (M/F)	5/16	11/11	10/17			
Age (years)	35.82 ± 10.91	39.91 ± 9.42	37.00 ± 7.93	0.146	0.653	0.264
BMI (kg/m^2^)	22.78 ± 1.62	28.05 ± 1.46	34.05 ± 3.50	<0.001	<0.001	<0.001
Height (cm)	162.78 ± 7.11	166.78 ± 8.85	162.82 ± 7.15	0.086	0.986	0.067
Weight (kg)	60.54 ± 7.56	78.18 ± 9.10	90.38 ± 12.05	<0.001	<0.001	<0.001
Waist Circumference (cm)	80.54 ± 6.08	93.47 ± 6.75	106.72 ± 8.28	<0.001	<0.001	<0.001
Hip Circumference (cm)	96.25 ± 4.61	106.12 ± 5.07	116.58 ± 9.21	<0.001	<0.001	<0.001
Systolic BP (mmHg)	114.77 ± 12.86	116.62 ± 10.26	117.83 ± 10.87	0.585	0.338	0.701
Diastolic BP (mmHg)	71.92 ± 10.88	72.97 ± 8.86	75.00 ± 7.94	0.703	0.235	0.427
Body Fat%	26.25 ± 5.16	32.40 ± 7.33	40.86 ± 7.55	0.004	<0.001	<0.001
Total Cholesterol (mg/dL)	177.06 ± 39.69	189.51 ± 47.25	181.54 ± 30.52	0.345	0.647	0.461
HDL (mg/dL)	52.56 ± 11.46	41.38 ± 10.22	38.67 ± 7.82	0.001	<0.001	0.279
LDL (mg/dL)	107.50 ± 36.39	115.92 ± 31.55	117.93 ± 27.21	0.411	0.242	0.804
Triglycerides (mg/dL)	85.03 ± 44.69	175.93 ± 228.04	124.65 ± 62.77	0.073	0.015	0.244
Glucose (mg/dL)	91.18 ± 7.44	97.14 ± 22.15	93.01 ± 8.80	0.237	0.435	0.355
Insulin (mIU/mL)	5.60 ± 3.59	6.86 ± 4.17	12.42 ± 6.22	0.298	<0.001	0.001
HOMA-IR (units)	1.28 ± 0.84	1.64 ± 1.07	2.85 ± 1.42	0.301	<0.001	<0.001

**Table 2:** Men’s macronutrient intake.

Men’s Intake	Intake Data			*p*-Value		
	Normal	Overweight	Obese	Normal vs. Overweight	Normal vs. Obese	Overweight vs. Obese
**Total Calories (kcal/day)**	2849 ± 1416	2593 ± 1123	3888 ± 2784	0.7328	0.3559	0.1958
**Protein (g)**	130 ± 55	105 ± 41	168 ± 129	0.3870	0.4437	0.1667
**Total Fat (g)**	106 ± 64	88 ± 34	131 ± 89	0.5643	0.5510	0.1759
**Carbohydrates (g)**	341 ± 155	351 ± 200	525 ± 401	0.9139	0.2261	0.2382
**Fiber (g)**	29 ± 15	28 ± 17	42 ± 34	0.9270	0.3297	0.2701

Data are represented by mean ± SD; *p* < 0.05 represents a significant difference.

**Table 3:** Women’s macronutrient intake.

Women’s Intake	Intake Data			*p*-Value		
	Normal	Overweight	Obese	Normal vs. Overweight	Normal vs. Obese	Overweight vs. Obese
**Total Calories (kcal/day)**	1920 ± 683	1938 ± 976	3326 ± 1295	0.9585	**0.0006**	**0.0034**
**Protein (g)**	82 ± 31	80 ± 38	140 ± 56	0.8371	**0.0011**	**0.0022**
**Total Fat (g)**	61 ± 23	64 ± 29	123 ± 50	0.7969	**0.0001**	**0.0005**
**Carbohydrates (g)**	268 ± 96	265 ± 152	429 ± 178	0.9494	**0.0033**	**0.0157**
**Fiber (g)**	24 ± 10	24 ± 10	38 ± 19	0.8605	**0.0172**	**0.0176**

Data are represented by mean ± SD; *p* < 0.05 represents a significant difference and is bolded.

**Table 4:** Two-way ANOVA assessing sex, BMI, and the metabolome.

BMI		Sex	
Metabolites	*p*-Value	Metabolites	*p*-Value
Acetohydroxamic acid	0.009	Glucose/Galactose	0.005
TMAO	0.014	Succinate	0.032
Acetylcarnitine	0.004		
Asparagine	0.018		
Creatinine	0.003		
Glutamic acid	0.028		
Pipecolic acid	0.002	**Sex and BMI**	
Cytidine	0.048	**Metabolites**	***p*-Value**
Leucic acid	0.044	Fructose	0.021
D-Galacturonic acid	0.042	Glyceric acid	0.039
Picolinic acid	0.044	Pregnenolone sulfate	0.016
2-Pyrrolidinone	0.007	Acetylornithine	0.046
Kynurenine	0.032	Phenylpyruvic acid	0.023
Nonadecanoic acid	0.012		
Decanoylcarnitine	0.035		
2-Aminoadipic acid	0.019		

**Table 5:** Corrected MANOVA assessing caloric intake and the metabolome.

Corrected Caloric Intake Model	
Metabolites	*p*-Value
***Men***	
Methylguanidine	0.004
2-Hydroxyphenylacetic acid	0.035
***Women***	
Stearic acid	0.002
Nonadecanoic acid	0.011
Malic acid	0.026
Dimethylglycine	0.032

## Data Availability

The mass spectrometry metabolomics data were deposited to the Mendeley Data Archive (http://dx.doi.org/10.17632/fby2jdy7m2.1 (accessed on 5 April 2021)) with the dataset identifier doi:10.17632/fby2jdy7m2.1.

## Supplementary Materials

The following are available online at https://www.mdpi.com/article/10.3390/metabo11080552/s1: Figure S1: Pie charts of macronutrient breakdown between sex and BMI groups, Figure S2: Significant metabolites by mean abundances of BMI & sex classification for men and women, Figure S3: Correlation heatmap of significant metabolites and subject demographics performed using Pearson’s *r*. All subjects included in the analysis, correlation cutoff = 0.5. Figure S4: Enrichment Analysis assessing the impact of sex on the metabolome, Table S1: A complete breakdown of men’s micronutrient and macronutrient intake, Table S2: A complete breakdown of women’s micronutrient and macronutrient intake, Table S3: Men’s food group consumption between normal, overweight, and obese subjects, Table S4: Women’s food group consumption between normal, overweight, and obese subjects, Table S5: Significant metabolites by BMI classification for men and women, Table S6: Matrix of correlation analysis between metabolites showing *r* and (*p*-value), Table S7: Pathway analysis of the impact of sex on the metabolome, Table S8: Fold changes and *p*-values of significant metabolites between men and women.
