# Supplementary material for: A Metabolomic Analysis of the Sex-Dependent Hispanic Paradox
Source: Metabolites. 2021 Aug 20;11(8):552. doi: 10.3390/metabo11080552 (PMC8401672; doi:10.3390/metabo11080552)
Supplement: Supplementary file 1 [file metabolites-11-00552-s001.zip › metabolites-1243283-supplementary.pdf]

Table S1. A complete breakdown of men's micronutrient and macronutrient intake.

Men's Nutrient Intake Profile	Intake Data			*P-value*		
	Normal	Overweight	Obese	Normal vs. Overweight	Normal vs. Obese	Overweight vs. Obese
**Calories (kcal)**	2849.22±1416.42	2593.05±1122.78	3887.58±2783.63	0.7328	0.3559	0.1958
**Protein (g)**	130.43±54.86	104.87±40.76	167.99±128.71	0.3870	0.4437	0.1667
**Tryptophan (g)**	1.31±0.5	1.07±0.43	1.6±1.26	0.3764	0.5360	0.2289
**Threonine (g)**	4.5±1.64	3.51±1.26	5.89±4.61	0.2750	0.4086	0.1438
**Isoleucine (g)**	5.53±2.24	4.26±1.56	6.95±5.37	0.2970	0.4830	0.1564
**Leucine (g)**	10.03±4.17	7.59±2.84	12.05±9.02	0.2814	0.5642	0.1640
**Lysine (g)**	8.31±3.28	6.08±2.16	10.46±8.44	0.2163	0.4944	0.1419
**Methionine (g)**	2.7±1.05	2.06±0.75	3.43±2.71	0.2694	0.4674	0.1531
**Cysteine (g)**	1.89±1.12	1.48±0.63	2.12±1.59	0.4685	0.7588	0.2571
**Phenylalanine (g)**	5.42±2.19	4.32±1.67	6.71±5.03	0.3535	0.5019	0.1798
**Tyrosine (g)**	4.23±1.81	3.23±1.21	5.16±4	0.3082	0.5446	0.1723
**Valine (g)**	6.32±2.59	4.88±1.83	7.82±5.99	0.3076	0.5104	0.1658
**Arginine (g)**	6.46±2.42	5.03±1.75	8.51±6.83	0.2819	0.4123	0.1488
**Histamine (g)**	3.41±1.28	2.58±0.94	4.24±3.25	0.2412	0.4928	0.1492
**Alanine (g)**	5.88±2.24	4.48±1.55	7.95±6.28	0.2534	0.3703	0.1201
**Aspartic acid (g)**	10.67±4.31	8.23±2.95	13.6±10.97	0.2964	0.4726	0.1639
**Glutamic acid (g)**	22.47±9.72	18.8±7.58	28.27±20.91	0.4818	0.4760	0.2023
**Glycine (g)**	4.95±2.09	4±1.38	7.02±5.52	0.3889	0.3161	0.1233
**Proline (g)**	8.06±4.05	6.69±2.77	9.94±7.01	0.5205	0.5230	0.1967
**Serine (g)**	5.64±2.42	4.44±1.73	6.91±5.19	0.3548	0.5284	0.1786
**Hydroxyproline (g)**	0.29±0.27	0.17±0.06	0.33±0.37	0.3787	0.8009	0.1992
**Carbohydrate (g)**	340.68±154.57	350.84±199.65	524.63±401.48	0.9139	0.2261	0.2382
**Glucose (g)**	15.3±8.07	17.5±9.21	43.51±66.58	0.6404	0.2175	0.2508
**Fructose (g)**	16.91±8.57	20.57±10.61	48.56±71.47	0.4814	0.1991	0.2501
**Galactose (g)**	0.14±0.11	0.12±0.06	0.19±0.17	0.6623	0.5323	0.2277
**Sucrose (g)**	28.57±21.22	26.21±17.63	35.42±30.75	0.8353	0.6237	0.4201
**Lactose (g)**	31.31±46.12	20.52±15.6	17.94±24.09	0.6344	0.5690	0.7770
**Maltose (g)**	0.87±0.6	0.71±0.22	2.03±2.11	0.5943	0.1319	0.0793
**Starch (g)**	47.6±23.79	38.62±18.86	59.67±43.53	0.4829	0.5004	0.1829
**Fiber (g)**	28.85±15.2	28.05±16.52	41.8±34.24	0.9270	0.3297	0.2701
**Total Fat (g)**	106.24±63.79	87.57±33.78	130.94±89.35	0.5643	0.5510	0.1759
***Trans* fat (g)**	2.19±2.66	1.39±0.57	1.99±1.5	0.5418	0.8796	0.2626
***Trans*-MUFA (g)**	1.47±1.8	0.99±0.42	1.46±1.15	0.5873	0.9908	0.2516
***Trans*-PUFA (g)**	0.37±0.51	0.2±0.09	0.29±0.25	0.4948	0.7347	0.3317
**Saturated Fat (g)**	32.89±19.11	28.77±12.08	41.9±29.41	0.6743	0.4893	0.2135
**Butyric acid (g)**	0.68±0.48	0.62±0.4	0.81±0.67	0.7973	0.6794	0.4393
**Caproic acid (g)**	0.37±0.25	0.36±0.25	0.44±0.35	0.9037	0.7104	0.5749
**Caprylic acid (g)**	0.28±0.17	0.29±0.21	0.34±0.33	0.8892	0.6170	0.6803
**Capric acid (g)**	0.49±0.32	0.49±0.29	0.6±0.5	0.9956	0.6123	0.5444
**Lauric acid (g)**	0.61±0.43	0.76±0.68	0.9±0.89	0.6068	0.4122	0.6904
**Myristic acid (g)**	2.62±1.53	2.36±1.27	3.31±2.68	0.7458	0.5376	0.3232
**Palmitic acid (g)**	18.46±10.44	15.67±6.35	23.42±16.09	0.6015	0.4866	0.1802
**Stearic acid (g)**	8.2±5.09	7.38±3.07	10.84±7.59	0.7517	0.4409	0.2042
**Arachidic acid (g)**	0.22±0.17	0.15±0.08	0.21±0.14	0.4421	0.8738	0.3071
**Behenic acid (g)**	0.21±0.24	0.14±0.13	0.16±0.17	0.5662	0.6727	0.7953
**MUFA (g)**	42.65±25.16	34.96±12.84	50.81±33.89	0.5469	0.6104	0.1911
**Myristoleic acid (g)**	0.12±0.1	0.09±0.04	0.1±0.07	0.4642	0.6792	0.5801
**Palmitoleic acid (g)**	1.61±0.82	1.31±0.51	2.16±1.52	0.4781	0.3764	0.1181
**Erucic acid (g)**	0.04±0.07	0.05±0.09	0.09±0.21	0.8191	0.5262	0.6005
**Gadoleic acid (g)**	0.33±0.2	0.27±0.12	0.38±0.26	0.5246	0.7178	0.2426
**Oleic acid (g)**	38.8±22.82	32.02±11.81	46.57±31.15	0.5580	0.5948	0.1915
**Elaidic acid (g)**	10.42±7.65	6.96±2.83	9.75±6.02	0.3758	0.8687	0.2049
**Vaccenic acid (g)**	1.3±1.64	0.96±0.5	1.16±1.1	0.6779	0.8732	0.6124
**Rumenic acid (g)**	0.04±0.03	0.02±0.01	0.03±0.03	0.2846	0.9631	0.0741
**PUFA (g)**	23.23±16.23	17.36±7.1	27.41±19.42	0.4753	0.66952	0.1500
**Linoleic acid (g)**	20.54±14.74	15.29±6.31	23.98±17.12	0.4806	0.6962	0.1576
**Linolenic acid (g)**	1.98±1.05	1.57±0.67	2.58±1.85	0.4639	0.4358	0.1326
**Arachidonic acid (g)**	0.2±0.05	0.16±0.09	0.26±0.2	0.2535	0.4050	0.1741
**DPA (g)**	0.01±0	0.01±0.01	0.02±0.03	0.9730	0.4321	0.4428
**EPA (g)**	0.02±0.01	0.02±0.01	0.04±0.06	0.8276	0.4708	0.5093

**DHA (g)**	0.07±0.06	0.05±0.03	0.13±0.23	0.5489	0.4659	0.3303
**Cholesterol (mg)**	473.65±93.69	367.07±200.98	536.97±390.65	0.1702	0.6370	0.2385
**Stigmasterol (mg)**	6.52±5.39	4.21±2.71	7.42±5.06	0.4068	0.7643	0.0970
**Campesterol (mg)**	16.26±6.33	10.88±4.51	17.91±8.79	0.1378	0.6850	0.0404
**Daidzein (mg)**	0.21±0.14	0.12±0.05	0.17±0.14	0.2265	0.6315	0.3041
**Genistein (mg)**	0.17±0.13	0.12±0.06	0.15±0.13	0.4863	0.8062	0.5497
**Caffeine (mg)**	198.7±189.28	83.57±101.24	122.5±128.21	0.2561	0.4483	0.4535
**Vitamin C (mg)**	141.54±75.42	137.44±54.12	365.14±457.02	0.9165	0.1630	0.1510
**Thiamin (mg)**	2.22±0.91	2.41±1.43	3.7±2.74	0.7549	0.1470	0.2034
**Riboflavin (mg)**	3.58±2.48	3.02±1.66	4.55±3.6	0.6643	0.5554	0.2437
**Niacin (mg)**	34.14±13.41	30.35±16.23	50.63±38.03	0.6358	0.2427	0.1445
**Pantothenic acid (mg)**	8.86±4.59	6.69±2.94	10.5±8.26	0.3733	0.6307	0.1956
**Pyridoxine (mg)**	3.17±1.28	2.72±1.37	5.24±4.45	0.5469	0.1986	0.1155
**Cobalamin (μg)**	10.82±7.98	10.42±9.93	12.6±10.86	0.9336	0.7253	0.6371
**Choline (mg)**	547.94±227.67	431.35±170.17	690.01±583.89	0.3451	0.5126	0.2058
**Folic acid (μg)**	201.95±58.97	282.87±221.38	548.4±678.01	0.2806	0.1422	0.2625
**Folate (μg)**	355.32±172.62	323.51±168.99	612.87±742.9	0.7402	0.3205	0.2568
**Vitamin A (IU)**	8755.17±6155.35	8216±4851.85	25178.78±42184.34	0.8680	0.2564	0.2373
**Retinol (μg)**	952.38±848.97	901.65±940.88	877.07±852.74	0.9172	0.8755	0.9506
**RAE (μg)**	1244.65±979.47	1168.2±975.19	2012.48±2768.39	0.8885	0.4476	0.3802
***β*-carotene (μg)**	3138.56±2453.9	2766.77±1783.66	12516.57±23555.22	0.7712	0.2431	0.2238
**α-carotene (μg)**	516.09±216.24	595.73±550.67	1578±2090.27	0.6849	0.1450	0.1797
**Vitamin E (mg)**	11.38±6.78	8.37±3	16.92±16.81	0.3863	0.3827	0.1453
**Vitamin D (IU)**	400.41±492.74	289.24±194.16	334.95±391.05	0.6483	0.8035	0.7437
**Vitamin D2 (μg)**	0.01±0.01	0±0.01	0.02±0.03	0.5093	0.4221	0.2279
**Vitamin D3 (μg)**	9.36±12.12	6.17±3.82	7.32±9.41	0.5933	0.7518	0.7252
**Vitamin D (*μ*g)**	9.98±12.27	7.21±4.85	8.32±9.72	0.6480	0.8006	0.7479
**Cryptoxanthin (μg)**	152.42±61.4	157.71±100.05	498.45±768.89	0.8989	0.1902	0.1968
**Lycopene**	9727.87±7756.66	8131.87±4076.88	18930.63±32829	0.6825	0.4187	0.3279
**Lutein (μg)**	2337.18±1853.43	2372.47±1547.51	12382.28±26801.91	0.9715	0.2679	0.2684
**Betaine (μg)**	64.96±29.39	61.15±31.77	125.68±177.86	0.8203	0.3181	0.2857
***β*-tocopherol (mg)**	0.13±0.08	0.11±0.04	0.16±0.14	0.6144	0.6228	0.3001
***γ*-tocopherol (mg)**	7.91±5.34	6.36±3.26	10.12±7.82	0.5726	0.5336	0.1840
***δ*-tocopherol (mg)**	1.56±1.17	1.41±0.76	2.19±1.75	0.8017	0.4256	0.2167
**Vitamin K (μg)**	143.99±90.21	109.78±61.64	517.89±1132.65	0.4715	0.3260	0.2844
**Calcium (mg)**	1638.77±1318.06	1335.84±585.56	1993.93±1579.95	0.6445	0.6560	0.2396
**Iron (mg)**	21.81±9	22.72±14.22	40.82±32.48	0.8805	0.1120	0.1296
**Magnesium (mg)**	486.81±265.34	407.73±166.5	686.17±604.28	0.5639	0.3916	0.1884
**Phosphorus (mg)**	2319.14±1194.68	1819.66±787.33	2673.14±1991.9	0.4277	0.6756	0.2298
**Potassium (mg)**	4485.62±2460.57	3666.37±1529.95	6383.01±5808.11	0.5204	0.3918	0.1815
**Sodium (mg)**	4527.76±2041.31	4264±1983.85	6723.68±5124.26	0.8154	0.2593	0.1812
**Zinc (mg)**	18.76±9.66	15.45±6.19	26.18±19.85	0.5100	0.3478	0.1305
**Copper (mg)**	2.24±1.53	2.31±1.72	2.81±2.27	0.9346	0.5748	0.5803
**Manganese (mg)**	3.71±1.81	4.09±2.33	6.79±5.71	0.7296	0.1459	0.1896
**Selenium (μg)**	167.36±61.53	142.11±60.77	214.31±158.6	0.4677	0.4270	0.2026

Data are represented by mean±SD; $P < 0.05$ represents a significant difference and is bolded

Table S2. A complete breakdown of women's micronutrient and macronutrient intake.

Women's Nutrient Intake Profile	Intake Data			*P-value*		
	Normal	Overweight	Obese	Normal vs. Overweight	Normal vs. Obese	Overweight vs. Obese
**Calories (kcal)**	1920.13±682.75	1938.09±976.16	3325.58±1294.92	0.9585	**0.0006**	**0.0034**
**Protein (g)**	82.41±30.77	79.53±38.03	139.89±55.75	0.8371	**0.0011**	**0.0022**
**Tryptophan (g)**	0.83±0.34	0.82±0.39	1.41±0.59	0.9247	**0.0019**	**0.0038**
**Threonine (g)**	2.68±0.99	2.73±1.28	4.75±1.95	0.9202	**0.0007**	**0.0027**
**Isoleucine (g)**	3.34±1.27	3.29±1.66	5.81±2.3	0.9426	**0.0007**	**0.0024**
**Leucine (g)**	6±2.33	5.99±3.1	10.42±4.17	0.9956	**0.0008**	**0.0035**
**Lysine (g)**	4.79±1.77	4.8±2.51	8.51±3.57	0.9901	**0.0008**	**0.0034**
**Methionine (g)**	1.6±0.61	1.62±0.81	2.82±1.16	0.9528	**0.0008**	**0.0034**
**Cysteine (g)**	1.12±0.53	1.14±0.8	1.89±0.74	0.9493	**0.0019**	**0.0222**
**Phenylalanine (g)**	3.38±1.3	3.35±1.67	5.86±2.31	0.9567	**0.0008**	**0.0026**
**Tyrosine (g)**	2.59±1.01	2.53±1.28	4.52±1.84	0.8995	**0.0009**	**0.0024**
**Valine (g)**	3.91±1.45	3.84±1.96	6.77±2.73	0.9253	**0.0009**	**0.0028**
**Arginine (g)**	3.64±1.35	3.88±1.86	6.69±2.82	0.7120	**0.0005**	**0.0038**
**Histamine (g)**	1.98±0.76	2.03±0.99	3.51±1.49	0.8976	**0.0010**	**0.0039**
**Alanine (g)**	3.27±1.21	3.5±1.68	6.09±2.57	0.6988	**0.0005**	**0.0035**
**Aspartic acid (g)**	6.23±2.18	6.4±3.22	11.02±4.49	0.8841	**0.0007**	**0.0039**
**Glutamic acid (g)**	14.86±6.04	14.34±7.28	25.47±10.08	0.8452	**0.0010**	**0.0023**
**Glycine (g)**	2.76±1.04	3.08±1.51	5.53±2.99	0.5490	**0.0018**	**0.0084**
**Proline (g)**	5.54±2.33	5.12±2.77	9.59±3.78	0.6809	**0.0009**	**0.0013**
**Serine (g)**	3.5±1.33	3.41±1.76	6.1±2.35	0.8811	**0.0006**	**0.0020**
**Hydroxyproline (g)**	0.1±0.07	0.13±0.08	0.2±0.1	0.3875	**0.0038**	**0.0477**
**Carbohydrate (g)**	268.1±96.42	264.76±152.07	428.54±178.08	0.9494	**0.0033**	**0.0157**
**Glucose (g)**	14.83±8.99	14.54±9.14	22±14.97	0.9365	0.1050	0.1141
**Fructose (g)**	17.33±10.44	14.59±6.53	24.56±16.87	0.4103	0.1482	0.0389
**Galactose (g)**	0.07±0.03	0.1±0.12	0.15±0.08	0.4326	**0.0015**	0.2897
**Sucrose (g)**	16.68±10.98	21.3±30.76	30.75±20.5	0.6416	**0.0204**	0.3826
**Lactose (g)**	18.01±16.23	15.23±24.98	22.41±18.87	0.7492	0.4773	0.4261
**Maltose (g)**	0.61±0.36	1.14±1.4	1.15±0.55	0.2493	**0.0023**	0.9705
**Starch (g)**	37.45±18.5	33.92±26.97	49.53±17.24	0.7110	0.0621	0.1081
**Fiber (g)**	24.28±9.69	23.59±10.12	37.76±19.25	0.8605	**0.0172**	**0.0176**
**Total Fat (g)**	61.23±22.98	63.94±28.78	123.44±49.54	0.7969	**0.0001**	**0.0005**
***Trans* fat (g)**	0.96±0.36	1±0.6	2.18±1.27	0.8602	**0.0013**	**0.0029**
***Trans*-MUFA (g)**	0.68±0.27	0.7±0.41	1.62±1	0.8682	**0.0015**	**0.0028**
***Trans*-PUFA (g)**	0.12±0.05	0.14±0.1	0.3±0.18	0.5896	**0.0007**	**0.0043**
**Saturated Fat (g)**	21.26±9.11	21.11±10.26	42.6±18.74	0.9690	**0.0003**	**0.0006**
**Butyric acid (g)**	0.56±0.31	0.52±0.44	1.07±0.62	0.7867	**0.0061**	**0.0110**
**Caproic acid (g)**	0.31±0.2	0.27±0.22	0.54±0.33	0.6341	**0.0217**	**0.0152**
**Caprylic acid (g)**	0.24±0.17	0.2±0.13	0.39±0.24	0.4719	0.0511	**0.0115**
**Capric acid (g)**	0.42±0.23	0.37±0.28	0.73±0.41	0.6604	**0.0105**	**0.0096**
**Lauric acid (g)**	0.57±0.39	0.47±0.31	0.91±0.54	0.4678	**0.0413**	**0.0096**
**Myristic acid (g)**	2.01±1.09	1.83±1.03	3.77±1.94	0.6672	**0.0034**	**0.0020**
**Palmitic acid (g)**	11.28±4.54	11.54±5.45	22.8±9.56	0.8991	**0.0002**	**0.0005**
**Stearic acid (g)**	5.17±2.19	5.25±2.45	11.03±5.13	0.9309	**0.0003**	**0.0005**
**Arachidic acid (g)**	0.1±0.04	0.11±0.05	0.21±0.09	0.5508	**0.0002**	**0.0011**
**Behenic acid (g)**	0.08±0.06	0.09±0.05	0.15±0.08	0.6503	**0.0071**	**0.0166**
**MUFA (g)**	23.21±8.4	26.17±14.34	48.94±19.77	0.5473	**6.602E-05**	**0.0016**
**Myristoleic acid (g)**	0.06±0.03	0.07±0.08	0.13±0.06	0.7273	**0.0011**	**0.0590**
**Palmitoleic acid (g)**	0.9±0.37	1.06±0.56	1.92±0.79	0.4357	**0.0001**	**0.0024**
**Erucic acid (g)**	0.07±0.1	0.19±0.56	0.04±0.05	0.4830	0.4253	0.4091
**Gadoleic acid (g)**	0.18±0.08	0.22±0.19	0.33±0.15	0.4669	**0.0008**	0.1179
**Oleic acid (g)**	21.38±7.84	23.42±10.88	44.89±18.21	0.6016	**0.0001**	**0.0006**
**Elaidic acid (g)**	4.16±1.48	5.18±3.87	9.86±4	0.4225	**2.053E-05**	**0.0054**
**Vaccenic acid (g)**	0.5±0.24	0.61±0.61	1.37±0.95	0.5606	**0.0018**	**0.0164**
**Rumenic acid (g)**	0.02±0.01	0.02±0.02	0.04±0.02	0.7076	**0.0046**	**0.0245**
**PUFA (g)**	11.84±4.42	12.01±4.73	22.99±8.4	0.9263	**6.358E-05**	**0.0002**
**Linoleic acid (g)**	10.39±3.99	10.34±3.93	20.22±7.48	0.9754	**7.315E-05**	**0.0001**
**Linolenic acid (g)**	1.14±0.44	1.18±0.49	2.16±0.86	0.8276	**0.0002**	**0.0007**
**Arachidonic acid (g)**	0.1±0.05	0.12±0.08	0.19±0.09	0.4977	**0.0018**	**0.0449**
**DPA (g)**	0.01±0.01	0.01±0	0.01±0.01	0.2690	0.1815	**0.0435**
**EPA (g)**	0.02±0.01	0.02±0.02	0.03±0.02	0.8795	0.1049	0.1322
**DHA (g)**	0.04±0.02	0.03±0.02	0.07±0.05	0.5017	**0.0330**	**0.0136**

**Cholesterol (mg)**	234.32±120.08	271.48±157.3	467.48±212.24	0.5164	**0.0006**	**0.0096**
**Stigmasterol (mg)**	2.69±1.01	3.16±1.66	6.97±3.5	0.4190	**0.0001**	**0.0007**
**Campesterol (mg)**	7.75±3.49	9.58±4.69	16.87±8.52	0.2863	**0.0005**	**0.0074**
**Daidzein**	1.2±4.42	0.71±2.02	0.38±0.63	0.7003	0.4748	0.6159
**Genistein**	1.14±4.22	0.83±2.47	0.4±0.78	0.8104	0.5009	0.5911
**Caffeine**	86.13±118.15	76.38±122.67	86.53±107.83	0.8388	0.9920	0.8250
**Vitamin C (mg)**	114.1±48.49	131.81±87.76	216.58±192.92	0.5525	**0.0480**	0.1279
**Thiamin (mg)**	1.95±1.05	1.85±1.2	3.01±1.6	0.8358	**0.0306**	**0.0384**
**Riboflavin (mg)**	2.73±1.26	2.86±2.23	4.09±2.4	0.8710	0.0509	0.1786
**Niacin (mg)**	23.7±13.46	25.02±17.3	37.29±19.2	0.8333	**0.0249**	0.0926
**Pantothenic acid (mg)**	5.76±2.34	6.46±4.17	9.91±7.27	0.6234	**0.0373**	0.1229
**Pyridoxine (mg)**	2.6±1.71	2.51±1.81	3.97±3.32	0.8994	0.1441	0.1445
**Cobalamin (μg)**	8.98±8.06	18.19±26.29	16.54±18.61	0.2836	0.1405	0.8585
**Choline (mg)**	320.28±130.4	364.07±214.99	555.12±245.63	0.5552	**0.0020**	**0.0403**
**Folic acid (μg)**	330.94±420.44	204.34±198.65	403.95±340.13	0.3063	0.5890	0.0611
**Folate (μg)**	275.56±117.03	287.11±134.66	439.21±249.81	0.8199	**0.0232**	**0.0472**
**Vitamin A (IU)**	8568.65±7386.44	9927.23±8951.83	12872.29±11461.5	0.6825	0.2079	0.4543
**Retinol**	786.08±669.3	1661.5±2421.73	1356.06±1502.85	0.2671	0.1691	0.7134
**RAE**	1087±866.13	1883.43±2461.79	1780.89±1780.37	0.3238	0.1641	0.9064
**β-carotene**	3191.09±3124.96	2366.18±865.52	4449.92±4001.75	0.3297	0.3204	0.0526
**α-carotene**	629.73±926.45	360.81±255.23	825.98±811.34	0.2849	0.5234	**0.0393**
**Vitamin E**	7.53±3.96	6.67±2.85	13.27±8.97	0.5166	**0.0253**	**0.0104**
**Vitamin D (IU)**	239.95±157.01	225.55±257.32	322.59±212.86	0.8707	0.2126	0.3111
**Vitamin D2**	0.11±0.41	0.18±0.56	0.05±0.16	0.7470	0.5551	0.4648
**Vitamin D3**	5.24±4.19	4.69±6.18	7.16±4.95	0.8005	0.2378	0.2804
**Vitamin D (μg)**	5.99±3.96	5.59±6.31	8.02±5.33	0.8549	0.2231	0.3051
**Cryptoxanthin**	128.75±97.15	175.64±201.62	296±287.08	0.4863	0.0347	0.2044
**Lycopene**	6627.89±4404.32	6640.7±3617.68	14602.45±16412.12	0.9935	0.0690	0.0694
**Lutein**	2549.08±2060.93	1950.05±671.92	3153.87±3293.36	0.2926	0.5299	0.1613
**Betaine**	56.42±32.87	47.7±13.08	84.6±68.33	0.3495	0.1411	**0.0442**
**β-tocopherol**	0.07±0.03	0.07±0.03	0.14±0.06	0.9950	**0.0005**	**0.0007**
**γ-tocopherol**	3.95±1.89	3.92±1.82	8.19±3.28	0.9703	**0.0001**	**0.0002**
**δ-tocopherol**	0.83±0.45	0.75±0.31	1.96±1.02	0.6149	**0.0004**	**0.0002**
**Vitamin K**	132.96±106.45	107.94±22.36	180.53±165.77	0.3750	0.3324	0.0930
**Calcium**	1327.63±448.3	1118.5±793.39	2066.82±1190.16	0.4414	**0.0266**	**0.0178**
**Iron**	19.53±12.84	19.99±17.63	28.96±14.69	0.9428	**0.0584**	**0.1773**
**Magnesium**	356.42±112.14	338.67±165.53	535.33±252.55	0.7604	**0.0143**	**0.0195**
**Phosphorus**	1537.85±581.12	1438.61±777.14	2508.04±1128.03	0.7232	**0.0045**	**0.0064**
**Potassium**	3197.34±1123.15	3131.88±1662.87	5038.81±2384.65	0.9107	**0.0088**	**0.0195**
**Sodium**	3217.48±1360.2	3070.92±1584.84	5657.28±2179.03	0.8053	**0.0006**	**0.0012**
**Zinc**	12.79±5.37	12.66±6.52	21.61±13.1	0.9553	**0.0181**	**0.0243**
**Copper**	1.85±1.31	3.63±4.36	3.41±3.1	0.2133	0.0693	0.8872
**Manganese**	3.64±1.65	3.35±1.79	5.22±2.84	0.6677	0.0608	**0.0420**
**Selenium**	110.62±46.84	107.6±53.77	178.61±73.41	0.8814	**0.0036**	**0.0067**

Data are represented by mean±SD; $P < 0.05$ represents a significant difference and is bolded

Table S3. Men's food group consumption between normal, overweight, and obese subjects.

Men's Food Groups	Intake Data			*p*-value		
	Normal	Overweight	Obese	Normal vs. Overweight	Normal vs. Obese	Overweight vs. Obese
**Red Meat (g)**	107.21±88.89	84.19±39.49	165.78±188.82	0.6042	0.4289	0.2108
**White Meat (g)**	72.38±59.89	44.99±33.64	94.21±144.43	0.3813	0.6870	0.3178
**Poultry (g)**	54.34±56.1	32.46±27.14	51.99±56.28	0.4457	0.9409	0.3374
**Fish (g)**	16.35±24.72	9.07±8.44	41.44±92.6	0.5531	0.4393	0.2990
**Beans (g)**	105.4±123.94	92.75±136.7	45.53±26.31	0.8590	0.3433	0.2858
**Fruit (g)**	154.98±107.28	143.73±102.44	269.16±320.06	0.8489	0.3279	0.2616
**Fruit Juice (g)**	66.64±71.25	80.74±80.42	544.74±1305.85	0.7615	0.3358	0.3489
**Vegetables (g)**	151.68±105.09	176.04±120.08	550.91±1010.6	0.6911	0.2465	0.2730

Data are represented by mean±SD; $P < 0.05$ represents a significant difference and is bolded.

Table S4. Women's food group consumption between normal, overweight, and obese subjects.

Women's Food Groups	Intake Data			*p-value*		
	Normal	Overweight	Obese	Normal vs. Overweight	Normal vs. Obese	Overweight vs. Obese
**Red Meat (g)**	38.74±25.38	61.37±41.17	101.76±65.28	0.1251	0.0552	**0.00134**
**White Meat (g)**	39.57±20.78	30.78±18.95	56.33±37.48	0.2666	**0.0253**	0.1219
**Poultry (g)**	28.92±17.25	20.73±18.81	33.95±25.28	0.2636	0.1258	0.5076
**Fish (g)**	8.19±7.77	6.92±6.1	19.06±17.79	0.6378	**0.0170**	0.0314
**Beans (g)**	52.79±61.54	39.89±46.73	83.38±79.01	0.5421	0.2163	0.0747
**Fruit (g)**	210.64±140.15	176.11±139.38	270.7±227.19	0.5346	0.4175	0.2273
**Fruit Juice (g)**	62.21±88.97	133.17±95.96	136.18±178.48	0.3298	0.1662	0.9681
**Vegetables (g)**	202.56±202.37	246.87±146.85	281.27±203.58	0.5159	0.3317	0.6543

Data are represented by mean±SD; $P < 0.05$ represents a significant difference and is bolded

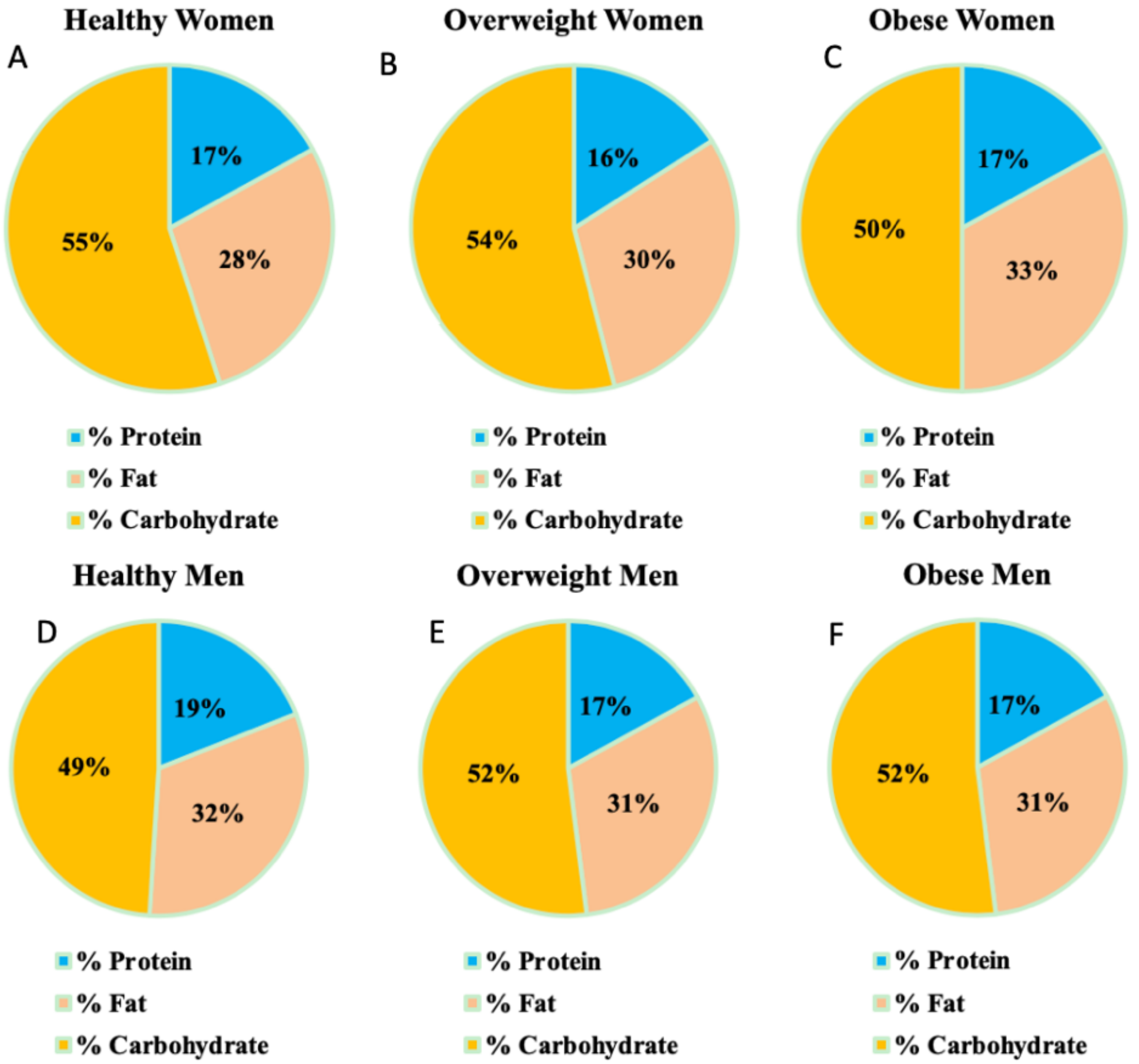

Figure S1. Pie charts of macronutrient breakdown between sex and BMI groups: (**A**) Normal weight women. (**B**) Overweight women. (**C**) Obese women. (**D**) Normal weight men. (**E**). Overweight men. (**F**) Obese men.

Table S5. Significant metabolites by BMI classification for men and women.

BMI			
**Metabolites**	**Post Hoc**		
	**Normal vs. Overweight**	**Normal vs. Obese**	**Overweight vs. Obese**
Acetohydroxamic acid			0.025
TMAO			0.038
Acetylcarnitine	0.017		
Asparagine	0.042		
Creatinine	0.011		0.013
Glutamic acid	0.006		0.01
Pipecolic acid	0.009		
Cytidine	0.015		0.016
Leucic acid			0.015
D-Galacturonic acid			0.012
Picolinic acid	0.008		0.045
2-Pyrrolidinone		0.002	0.016
Kynurenine	0.004		
Nonadecanoic acid			0.012
Decanoylcarnitine		0.026	
2-Aminoadipic acid	0.009		0.002

Sex	
**Metabolites**	***P*-Value**
Glucose/Galactose	0.005
Succinate	0.032

Key
Downregulated in Lower BMI Group or Men
Upregulated in Lower BMI Group or Men

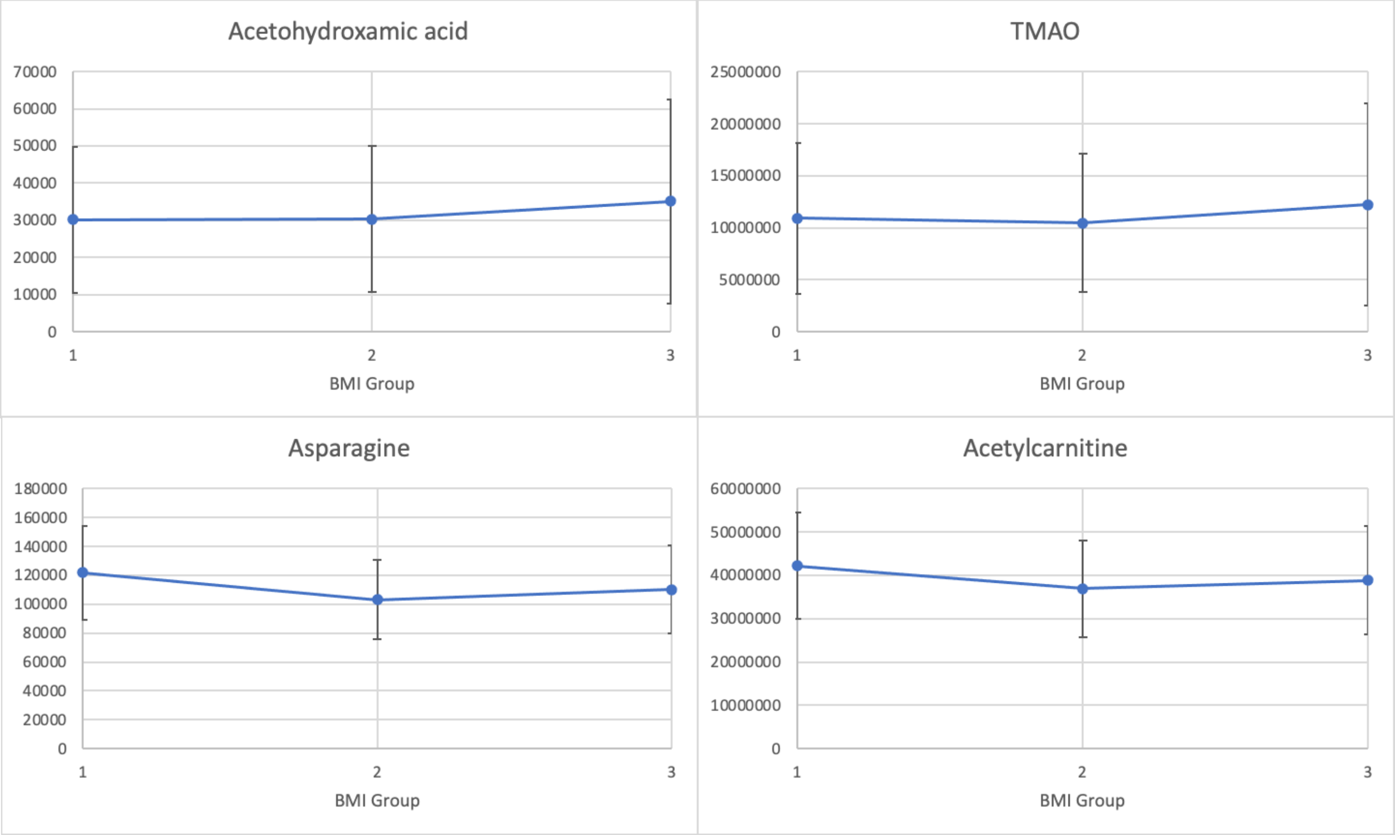

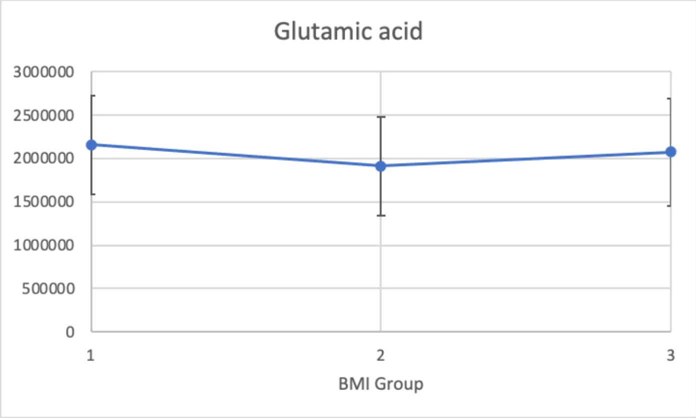
Glutamic acid
3000000
2500000
2000000
1500000
1000000
500000
0
1
2
3
BMI Group

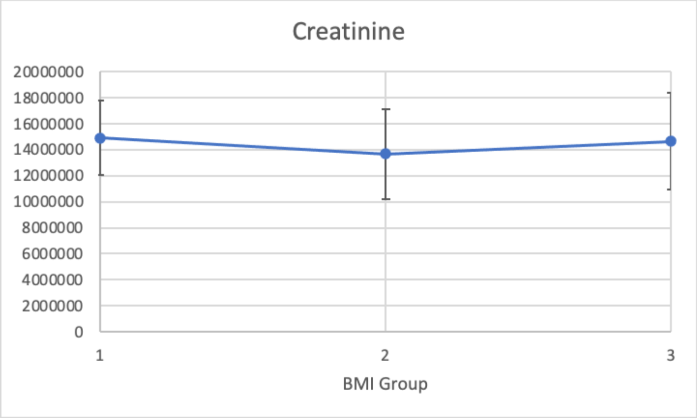
Creatinine
20000000
18000000
16000000
14000000
12000000
10000000
8000000
6000000
4000000
2000000
0
1
2
3
BMI Group

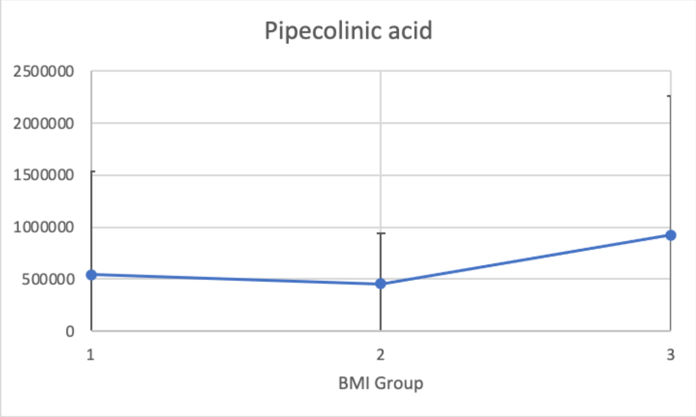
Pipecolinic acid
2500000
2000000
1500000
1000000
500000
0
1
2
3
BMI Group

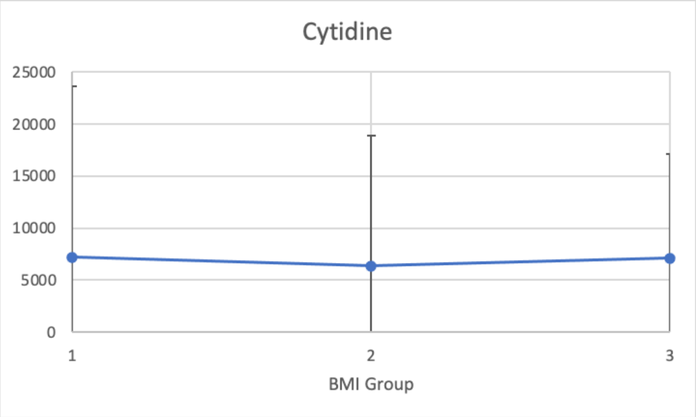
Cytidine
25000
20000
15000
10000
5000
0
1
2
3
BMI Group

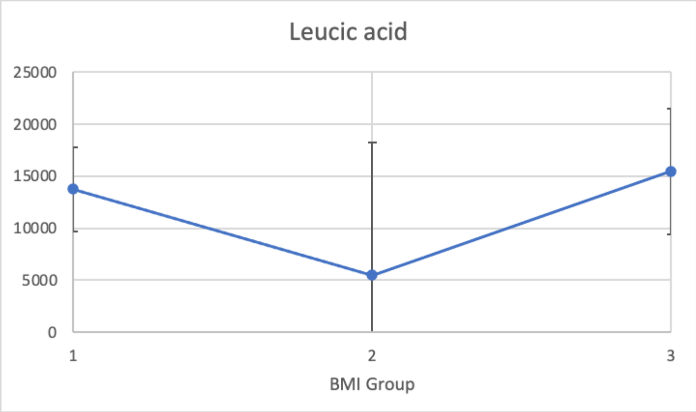
Leucic acid
25000
20000
15000
10000
5000
0
1
2
3
BMI Group

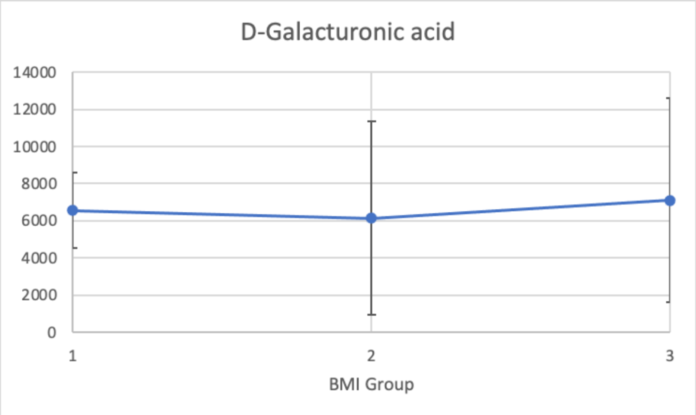
D-Galacturonic acid
14000
12000
10000
8000
6000
4000
2000
0
1
2
3
BMI Group

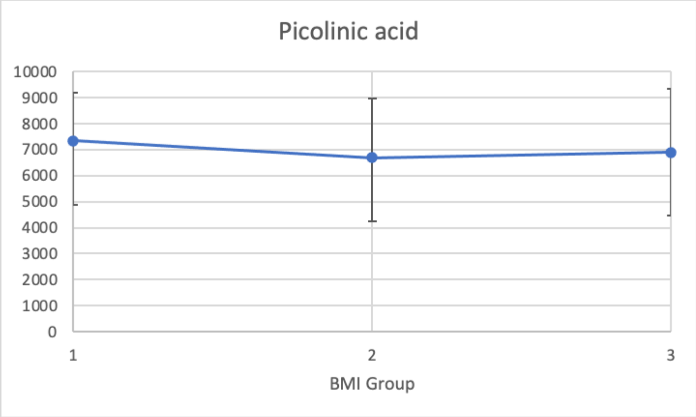
Picolinic acid
10000
9000
8000
7000
6000
5000
4000
3000
2000
1000
0
1
2
3
BMI Group

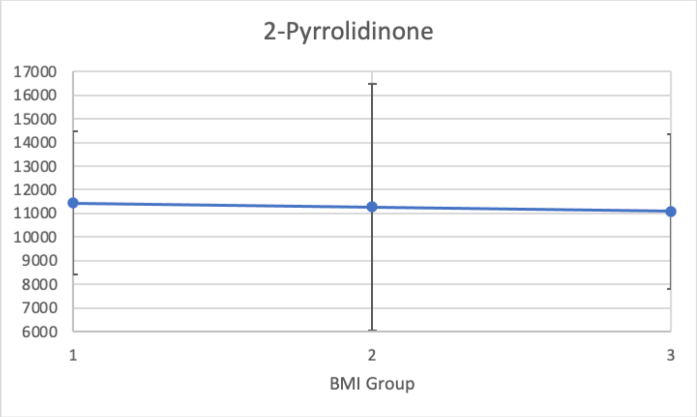
2-Pyrrolidinone
17000
16000
15000
14000
13000
12000
11000
10000
9000
8000
7000
6000
1
2
3
BMI Group

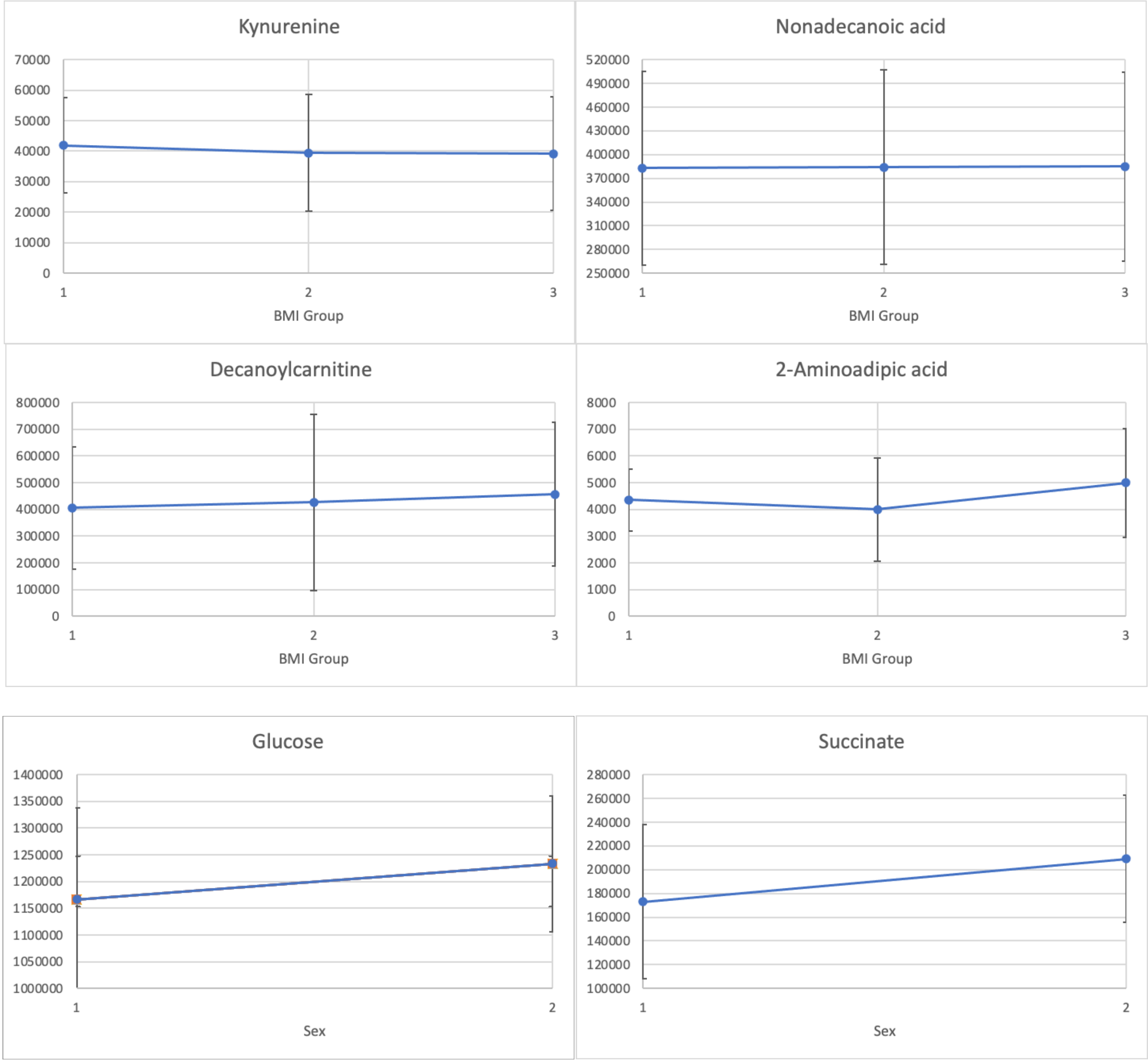

Figure S2. Significant metabolites by mean abundances of BMI & sex classification for men and women. BMI groups were stratified into three categories: 1 - normal weight (< 25 BMI), 2 - overweight (25 < BMI < 30), and 3 - obese (BMI > 30). Sex was classified as 1 - men and 2 – women.

Table S6. Matrix of correlation analysis between metabolites showing *r* and (*P*-Value).

Metabolites	Acetohydroxamic acid	Fructose	Isoleucine	Picolinic acid	2-Pyrrolidinone	Asparagine
**TMAO**	0.995 (<0.001)	0.116 (0.320)	0.028 (0.813)	0.095 (0.419)	0.277 (0.016)	-0.077 (0.509)
**Glucose**	0.150 (0.198)	0.879 (<0.001)	0.117 (0.319)	0.173 (0.137)	0.115 (0.327)	-0.024 (0.841)
**Kynurenine**	0.063 (0.593)	0.043 (0.713)	0.826 (<0.001)	0.684 (<0.001)	0.669 (<0.001)	0.606 (<0.001)
**Glutamic acid**	0.082 (0.484)	0.107 (0.359)	0.790 (<0.001)	0.518 (<0.001)	0.601 (<0.001)	0.644 (<0.001)
**Creatinine**	0.049 (0.678)	0.228 (0.049)	0.647 (<0.001)	0.533 (<0.001)	0.584 (<0.001)	0.513 (<0.001)
**2-Aminoadipic acid**	-0.045 (0.702)	0.097 (0.409)	0.451 (<0.001)	0.580 (<0.001)	0.313 (0.006)	0.228 (0.049)

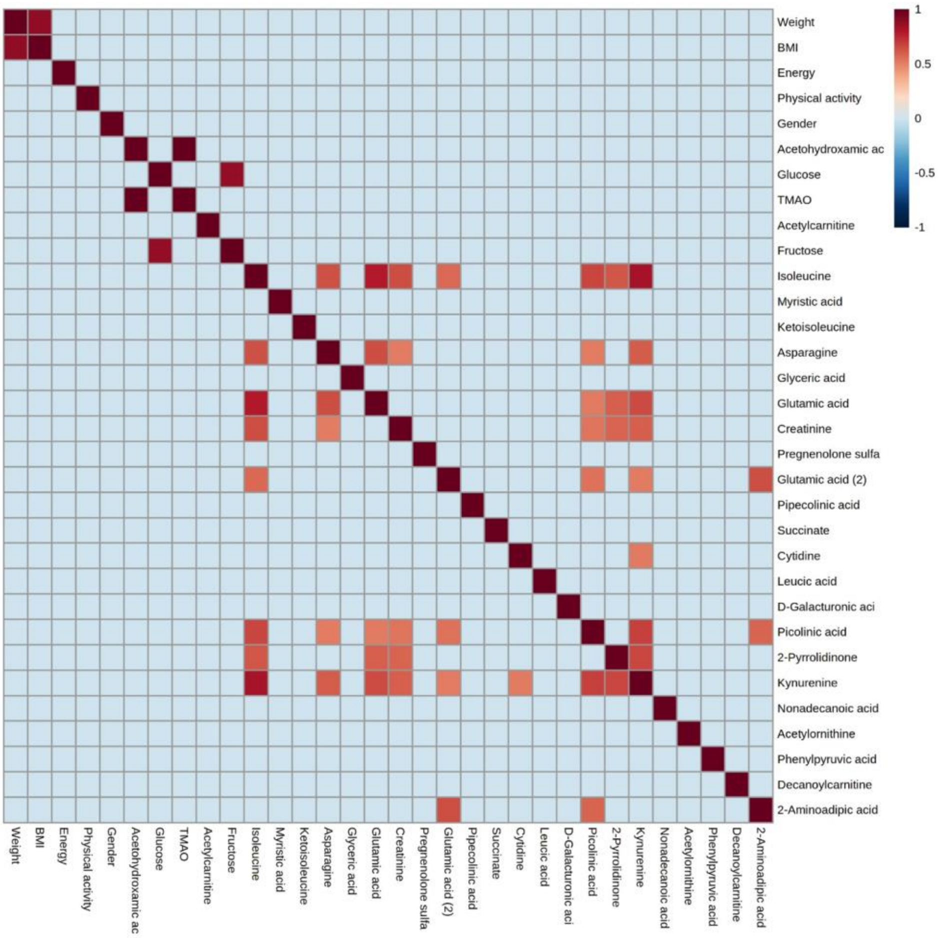

Figure S3. Correlation heatmap of significant metabolites and subject demographics performed using Pearson's *r*. All subjects included in the analysis, correlation cutoff = 0.5.

Table S7. Pathway Analysis of the impact of sex on the metabolome.

Pathway	Total Metabolites	*P*-Value	Impact
Lysine degradation	3	<0.001	0.14
Vitamin B_6 metabolism	1	0.012	0.0
Pantothenate and CoA biosynthesis	4	0.013	0.007
Tyrosine metabolism	3	0.018	0.14
Valine, leucine, and isoleucine degradation	5	0.018	0.021
Valine, leucine, and isoleucine biosynthesis	6	0.029	0.0
Pyruvate metabolism	3	0.046	0.292

Table S8. Fold changes and *P*-values of significant metabolites between men and women.

Metabolites	Fold Change (Women/men)	*P*-value
**Proline**	0.875	**0.015**
**Betaine**	0.88	**0.045**
**L-Alloisoleucine**	0.771	**0.009**
**Isoleucine**	0.747	**0.045**
**Myristic acid**	1.156	**0.024**
**2,3-Dihydroxybenzoic acid**	1.435	**0.022**
**3-Methyl-2-oxovaleric acid**	0.836	**0.001**
**Creatinine**	0.829	**0.001**
**Protocatechuic acid**	1.436	**0.021**
**Glutamic acid**	0.767	**0.007**
**Citraconic acid**	0.821	**0.001**
**Creatine**	1.185	**0.034**
**9-Octadecynoic acid**	1.122	**0.042**
**4-Methyl-2-oxopentanoic acid**	0.777	**< 0.001**
**2/3-Aminoisobutyric acid**	0.741	**0.001**
**Hypoxanthine**	0.763	**0.02**
**2-Hydroxyphenylacetic acid**	0.785	**< 0.001**
**Leucic acid**	0.744	**0.002**
**Picolinic acid**	0.841	**0.032**
**Lauric acid**	1.424	**< 0.001**
**Tyrosine**	0.828	**0.016**
**3-Phenyllactic acid**	0.726	**0.001**
**Phenylpyruvic acid**	0.841	**0.008**
**2-Aminoadipic acid**	0.689	**< 0.001**

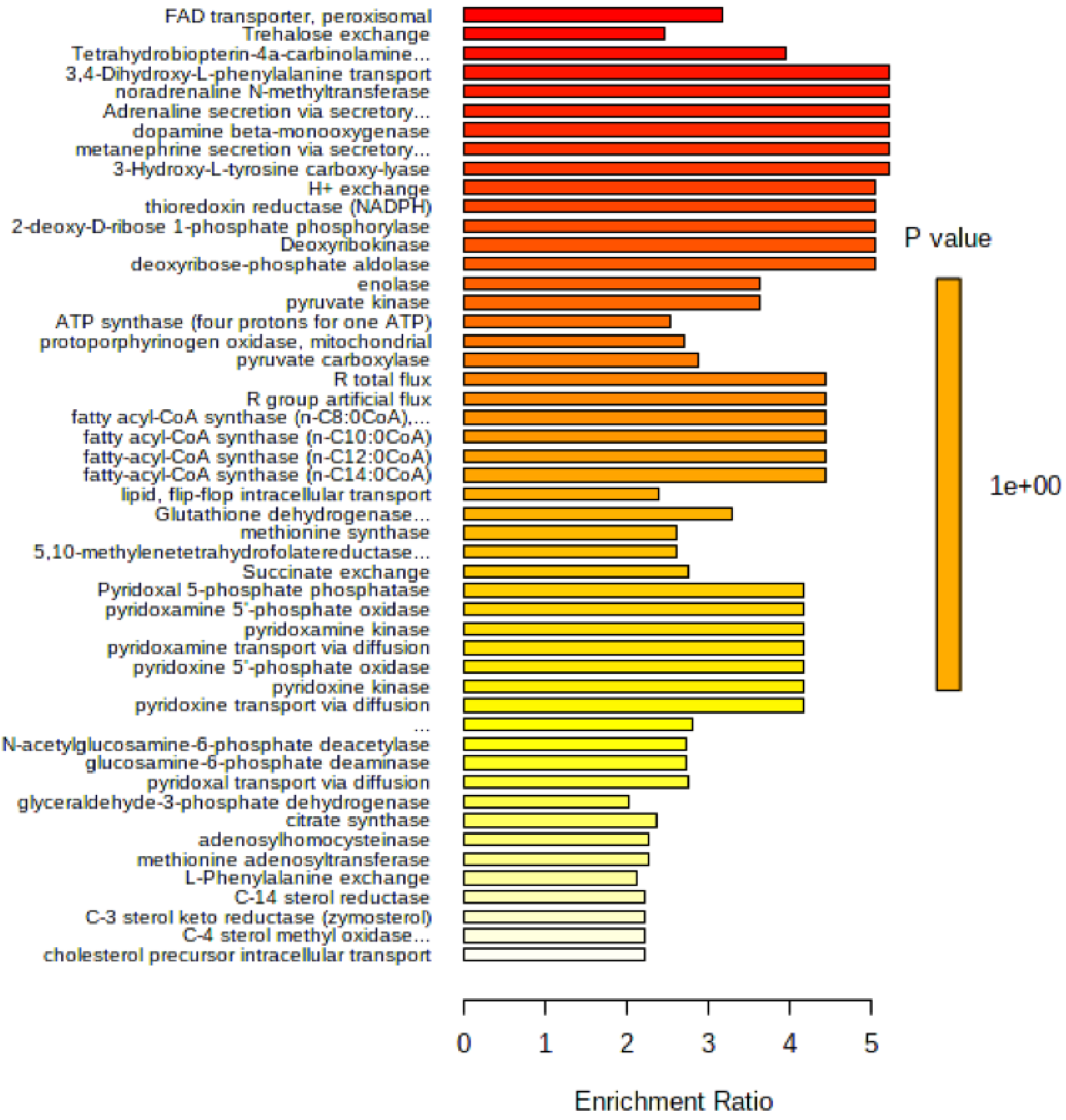

Figure S4. Enrichment Analysis assessing the impact of sex on the metabolome.
